# A Comparison of the Olfactory Gene Repertoires of Adults and Larvae in the Noctuid Moth *Spodoptera littoralis*

**DOI:** 10.1371/journal.pone.0060263

**Published:** 2013-04-02

**Authors:** Erwan Poivet, Aurore Gallot, Nicolas Montagné, Nicolas Glaser, Fabrice Legeai, Emmanuelle Jacquin-Joly

**Affiliations:** 1 INRA, UMR 1272, Physiologie de l’Insecte, Signalisation et Communication, Versailles, France; 2 IRISA, Équipe GenScale, Campus Universitaire de Beaulieu, Rennes, France; 3 UPMC - Université Paris 6, UMR 1272, Physiologie de l’Insecte, Signalisation et Communication, Paris, France; Plant and Food Research, New Zealand

## Abstract

To better understand the olfactory mechanisms in a lepidopteran pest model species, the cotton leafworm *Spodoptera littoralis*, we have recently established a partial transcriptome from adult antennae. Here, we completed this transcriptome using next generation sequencing technologies, namely 454 and Illumina, on both adult antennae and larval tissues, including caterpillar antennae and maxillary palps. All sequences were assembled in 77,643 contigs. Their analysis greatly enriched the repertoire of chemosensory genes in this species, with a total of 57 candidate odorant-binding and chemosensory proteins, 47 olfactory receptors, 6 gustatory receptors and 17 ionotropic receptors. Using RT-PCR, we conducted the first exhaustive comparison of olfactory gene expression between larvae and adults in a lepidopteran species. All the 127 candidate olfactory genes were profiled for expression in male and female adult antennae and in caterpillar antennae and maxillary palps. We found that caterpillars expressed a smaller set of olfactory genes than adults, with a large overlap between these two developmental stages. Two binding proteins appeared to be larvae-specific and two others were adult-specific. Interestingly, comparison between caterpillar antennae and maxillary palps revealed numerous organ-specific transcripts, suggesting the complementary involvement of these two organs in larval chemosensory detection. Adult males and females shared the same set of olfactory transcripts, except two male-specific candidate pheromone receptors, two male-specific and two female-specific odorant-binding proteins. This study identified transcripts that may be important for sex-specific or developmental stage-specific chemosensory behaviors.

## Introduction

In insects, both larvae and adults use their olfactory system to detect chemical cues in their environment, searching for food, for a mate or for adequate oviposition sites. In holometabolous insects, larvae and adults represent two morphologically different mobile forms with radically different physiologies and ecologies. The larvae feed, grow and accumulate energy in order to perform metamorphosis whereas the adults usually feed on different substrates and take over reproductive responsibilities. Accordingly, both forms are sensitive to different chemical cues. Thus, it is expected that their molecular equipment required for odor detection should be different. This has been verified in only a few model species, including the Diptera *Drosophila melanogaster* [1] and *Anopheles gambiae* [2] and the Lepidoptera *Bombyx mori* [3], species for which complete repertoires of olfactory genes have been described thanks to their sequenced genomes. These repertoires group several large families of proteins involved in different steps of odorant detection [4]. In the peripheral organs, odorant molecules first interact with binding proteins to cross the aqueous sensillum lymph to the olfactory receptor neurons (ORNs). Among these binding proteins, odorant-binding proteins (OBPs) and chemosensory proteins (CSPs) are proposed to bind general odorant compounds like host volatiles [5], [6], although the role of CSPs in chemoreception remains unclear. Pheromone-binding proteins (PBPs) consist of a subclass of OBPs and are proposed to specifically bind the sex pheromone components [7], [8]. After crossing the lymph, odorant molecules interact with receptors expressed in the ORN dentritic membrane. Two families of such receptors have been described in insects, the olfactory receptors (ORs) and the ionotropic receptors (IRs) [9], that are involved in the recognition of different volatile families in *D. melanogaster* [10], [11], [12]. ORs are seven-transmembrane domain receptors with an inverted membrane topology compared to G protein-coupled receptors [13]. They are specific to insects, they are very divergent between and within species, and are proposed to function as ion channels via heterodimerization with a subunit conserved within insects [13], [14], [15], [16], referred to as Orco [17]. Ligand spectra of large OR repertoires have been studied in detail only in *D. melanogaster* [18] and *A. gambiae* [19], [20], but numerous lepidopteran ORs specialized in the detection of sex pheromones –the so-called pheromone receptors (PRs) – have also been functionally characterized [21], [22], [23], [24], [25]. IRs constitute an evolutionary distinct family of chemosensory receptors and are far more ancient than ORs, as they are found across all protostomians [11]. They are related to ionotropic glutamate receptors but harbour a divergent ligand-binding domain [10]. Like ORs, they are supposed to function as ion channels, and form heterodimers with conserved co-receptors [26]. IRs have been first identified in *D. melanogaster* where they are notably involved in food odor detection [10], acid sensing [12] and in reproduction behaviour promotion [27].

In Lepidoptera, the olfactory organs of caterpillars and adults differ from each other. In larvae, they consist of a pair of small antennae and a pair of maxillary palps that, together, contain several decades of ORNs housed in a decade of olfactory sensilla [28], [29], [30], [31]. By contrast, adult antennae bear several thousands of olfactory sensilla that house two or three ORNs [32], [33], [34].

Whereas repertoires of genes encoding OBPs, CSPs, ORs and IRs have been described in diverse moths [3], [35], [36], [37], [38], [39] and butterflies [40], [41] through genome analyses or transcriptomic approaches, the expression of these genes in caterpillars has been investigated only for *B. mori* OBPs [36], CSPs [35] and ORs [3]. These studies revealed that caterpillars have a simpler olfactory system with a lower number of olfactory genes expressed and with some overlap between the two developmental stages, as observed in *Drosophila* and *A. gambiae* for ORs [1], [2], [42].

In the cotton leafworm *Spodoptera littoralis*, we have previously described the adult antennal transcriptome through the sequencing of expressed sequence tags (ESTs) from male and female antennae [38], [43]. Here, we took advantage of next generation sequencing technologies (NGS) to improve this transcriptome by re-sequencing adult antennae and sequencing larval tissues, including antennae and maxillary palps. All sequences obtained were assembled and their analysis greatly enriched the description of the olfactome in this crop pest species with a total of 127 candidate chemosensory genes, including 12 new *ORs*, 5 new *IRs* and 17 new *OBPs/CSPs*. With this repertoire in hand, we compared the expression of all these genes in male and female adult antennae, in adult and caterpillar olfactory organs, and in caterpillar antennae and maxillary palps. This investigation is the first to be conducted on a crop pest moth, and provides the molecular bases to better understand *S. littoralis* caterpillar olfaction.

## Materials and Methods

### Insect Rearing, 454 and Illumina Sequencing

*S. littoralis* were reared in the laboratory on a semi-artificial diet at 22°C, 60% relative humidity and under a 16∶8 light/dark cycle. Male and female antennae (200 from each sex) were dissected from 2-day-old adults. Other tissues (pool of adult proboscis and brains, larval head, whole body, gut, fat bodies and hemocytes) were also prepared to enrich the *S. littoralis* transcriptome. Antennae and maxillary palps (∼1000 each) were dissected from fourth instar larvae. Half of the larvae were starved for 24 h before dissection since starvation is known to enhance larval olfactory behaviour and starved larvae may express a different set of chemosensory genes than fed larvae. Total RNAs were extracted from each tissue using TRIzol® Reagent (Invitrogen, Carlsbad, CA, USA). Adult antennal RNAs were used as templates for cDNA synthesis and 454 sequencing (454 Roche GS FLX Titanium, ½ Pico Titer Plate for male antennae, ½ Pico Titer Plate for female antennae; LGC Genomics GmbH, Berlin, Germany). Pooled tissue RNAs were used as another template for 454 sequencing (454 Roche GS FLX Titanium, ½ Pico Titer; GATC Biotech SARL, Mulhouse, France). Both fed and starved larvae antennae and maxillary palp RNAs were used as templates for Illumina sequencing (one channel for the two samples, single read, HighSeq2000; GATC Biotech SARL). The data generated in this project have been deposited in GenBank (BioProject) under the accession numbers SAMN01908929 and SAMN01908927 (larvae antennae and palps, Illumina sequencing), SAMN01908931 (Female antennae, 454 sequencing), SAMN01908932 (Mixed tissues, 454 sequencing) and SAMN01908930 (Male antennae, 454 sequencing). All data were also included in LepidoDB (http://www.inra.fr/lepidodb/Spodoptera_littoralis), a centralized bioinformatic resource for the genomics of lepidopteran pests [44].

### Sequence Processing and Assembly

Sequence preprocessing was performed on 454 and Illumina data by removing adapters and by trimming low quality regions. Briefly, data were first analyzed with FastQC v. 0.10.0 (www.bioinformatics.babraham.ac.uk/projects/fastqc) that provided information on sequence quality and identified over-represented sequences within libraries. Over-represented sequences were removed with Cutadapt [45]. Then, sequences were trimmed to remove regions with low quality sequences with PRINSEQ v 0.17.3. [46]. Finally, sequences shorter than 20 bp long were removed from all data sets. After these preprocessing steps, a total of 1,375,379 sequences from 454 and 3,979,595 sequences from Illumina were kept for further analysis (Table 1). A first step of *de novo* assembly was performed on Illumina reads with Trinity release 2012-01-25 [47] with jellyfish method for k-mer counting. This first assembly step permited to reconstruct 11,560 contigs (>200 bp) from the two Illumina data sets. The final transcriptome assembly was performed on the two previously obtained adult EST data sets, the three 454 data sets and the Trinity assembly data set with the MIRA assembler v 3.2.1. using as parameters *de novo* assembly method, est assembly type, accurate quality, Sanger sequencing technology [48]. A total of 1,220,137 sequences were assembled into 77,643 contigs longer than 40 bp and containing at least two assembled sequences.

**Table 1:** Summary of data used for transcriptome assembly.

Sequencing technology	Sanger sequencing (EST)		454 sequencing			Illumina sequencing	
tissues	Male antennae	Female antennae	Male antennae	Female antennae	miscelaneous	fed larvae antennae and palps	starved larvae antennae and palps
Raw sequence number	–	–	557,390	656,772	424,199	1,947,899	2,389,809
Processed sequence number	20,760	18,342	530,329	430,760	414,290	1,807,931	2,171,664
Mean size (bp)	958	665	191	216	301	61	59
Size range (bp)	40–1525	72–888	31–550	31–574	30–580	20–72	20–72

### Transcriptome Annotation and Identification of Olfactory Genes

The obtained contigs were compared to the NCBI non redundant protein database (20.03.2012) using BLASTX, with a 1e−8 value threshold. The Gene Ontology mapping and annotation were done with BLAST2GO (GO association done by a BLAST against the NCBI NR database) [49]. Contigs were translated to peptides using FrameDP 1.2.0 [50] with three training iterations and using Swissprot (398.181, August 2009) as the reference protein database. GO annotation was then completed with Interproscan annotation of translated peptides. Within the newly generated *S. littoralis* transcriptome, olfactory transcripts were searched with available lepidopteran OBP, CSP, OR and IR amino acid sequences (see Phylogenetic analyses) as queries using TBLASTN. Sequences matching with the queries were further assembled using the Cap3 programme [51], when possible, to obtain longer contigs. Resulting contigs and singletons were reversely compared to NCBI NR database using the BLASTX application. Sequences whose best BLASTX hits corresponded to OBPs, CSPs, ORs and IRs were then retained as candidate *S. littoralis* olfactory transcripts and their translation was manually verified or corrected. These sequences were compared to the sequences of the already described olfactory genes in this species [38], [43] to identify novel genes. Novel OBPs/CSPs were searched for the presence of a signal peptide using SignalP 4.0 [52] and transmembrane domains of novel candidate ORs were predicted using the TMHMM server v.2.0 [53].

### RACE-PCR

Short sequences of new putative *ORs* were extended by rapid amplification of cDNA ends (RACE-PCR). cDNAs were synthesized from 1 µg of male antennal RNA at 42°C for 1.5 h, with SuperScript™ II reverse transcriptase (200 U, Gibco BRL, Invitrogen), using the 3′-CDS primer (for 3′RACE) or the 5′-CDS primer and the SMART™ II oligonucleotide (for 5′RACE), supplied in the SMART™ RACE cDNA amplification kit (Clontech), following the manufacturer’s instructions. RACE-PCRs were conducted using the Advantage™ 2 polymerase mix (Clontech) and the Universal Primer Mix versus the following gene-specific primers: 5′RACE primer OR46 5′- AAGCTGGATCTTCGGGACAGTTCATCA -3′; 3′RACE primer OR47 5′- TGATGAACTGTCCCGAAGATCCAGCTT -3′; 5′RACE primer OR46 5′- TCATACACCGCGTCTGCTACACCTACG -3′. Touchdown PCRs were performed following the manufacturer’s instructions with a final elongation step of 10 min at 72°C. The PCR products were cloned into the pCR®II-TOPO® plasmid (Invitrogen). Recombinant plasmids were isolated by mini preparation (QIAprep Spin Miniprep Kit, Qiagen), and both strands were sequenced (Biofidal, France).

### Phylogenetic Analyses

In addition to the sequences described in *S. littoralis*, the OR data set contained the complete or nearly complete amino acid sequences from the moths *Bombyx mori* [3], *Cydia pomonella* [39], *Heliothis virescens* [54], [55] and *Manduca sexta* [37], and also from the butterflies *Danaus plexippus* [40] and *Heliconius melpomene* [41]. It has to be noticed that some of the SlitOR sequences were short (see Results section) that may affect the accuracy of the phylogenetic analysis. The data set contained 261 sequences.

The OBP data set contained 191 amino acid sequences from *S. littoralis*, *B. mori* [36], *H. melpomene* [41], *H. virescens* [56], [57], [58], *M. sexta* [37], [59] and from three other species of the genus *Spodoptera* (*S. exigua*, *S. frugiperda* and *S. litura*) retrieved from GenBank: *S. exigua* ABP (ADY17881), GOBP1 (ACY78412), GOBP2 (CAC12831), OBP1 (ADY17882), OBP2 (ADY17883), OBP3 (ADY17884), OBP4 (ADY17885), OBP5 (AFM77983), OBP6 (AFM77984), OBP7 (ADY17886), PBP1 (AAU95536), PBP2 (AAU95537) and PBP3 (ACY78413); *S. frugiperda* GOBP2 (AAT74555), OBP1 (AAR28762) and OBP2 (AAR28763); *S. litura* GOBP1 (ABM54823), GOBP2 (ABM54824), PBP1 (AAY21255), PBP2 (AAZ22339) and PBP3 (ACY78414). Signal peptide sequences were removed from the data set.

The CSP data set contained 103 sequences from *S. littoralis*, *B. mori* [35], *H. melpomene* [41], *H. virescens* [60], *Papilio xuthus* [61] and *S. exigua* [62]. As for OBPs, signal peptide sequences were removed.

Since IRs are well conserved in insects, the IR data set contained sequences from the Lepidoptera *S. littoralis* ([63] and this study), *B. mori* [11], *C. pomonella* [39] and *D. plexippus* [40] but also from model insects: *D. melanogaster*, *Apis mellifera* and *Tribolium castaneum* [11].

Amino acid sequences were aligned with MAFFT v.6 [64] using the FFT-NS-2 algorithm and default parameters, except for the OR sequences that were aligned using Muscle [65] as implemented in Seaview v.4 [66]. All alignments were curated manually to remove highly divergent regions. Phylogenetic reconstructions were carried out using maximum likelihood. For each data set, the LG+I+G substitution model [67], was determined as the best-fit model of protein evolution by ProtTest 1.3 [68] following Akaike information criterion. Rate heterogeneity was set at four categories, and the gamma distribution parameter and the proportion of invariable sites were estimated from the data set. Tree reconstruction was performed using PhyML 3.0 [69], with both SPR (Subtree Pruning and Regrafting) and NNI (Nearest Neighbour Interchange) methods for tree topology improvement. Branch support was estimated by approximate likelihood-ratio test (aLRT) [70]. Images were created using the iTOL web server [71].

### RT-PCR

Total RNAs of two-day-old *S. littoralis* male and female antennae and 18 to 20-day-old larvae antennae and maxillary palps were extracted using TRIzol® Reagent (Invitrogen, Carlsbad, CA, USA). After a DNase I treatment (Promega, Madison, WI, USA), RNAs (0.75 µg) were used as templates for single stranded cDNA synthesis using the Advantage RT-for-PCR kit (Clontech, Mountain View, USA). For controls, RNAs were submitted in parallel to the same reactions except reverse transcriptase was omitted. PCRs were performed on the four tissues under the following conditions: 94°C for 1 min, 35 cycles of (94°C for 30 s, 53–64°C - depending on primer pairs - for 30 s, 72°C for 3 min) and 72°C for 10 min as a final extension step, using Titanium Taq DNA polymerase (Clontech) and with specific primer pairs designed for 127 *S. littoralis* olfactory genes. These genes consisted of 47 candidate *ORs* including 4 *PRs*, 36 *OBPs* including 3 *PBPs*, 21 *CSPs*, 17 *IRs* and 6 gustatory receptors (*GRs*). Five of these *GRs* were previously described in *S. littoralis* adult antennae [38], [43] and the sixth *GR* was newly identified in this study. Primer pairs were designed from the nucleotide sequences using the primer3+ software (http://www.bioinformatics.nl/cgi-bin/primer3plus/primer3plus.cgi). All primer sequences, annealing temperatures and expected product sizes are listed in Supporting Information S1. The ribosomal protein L8 gene (*rpL8*) was used as a RNA extraction control for the four tissues. For each amplification, negative controls consisted of amplifications run on DNase-treated RNAs and water templates. The amplification products were loaded on 1.5% agarose gels and visualized using ethidium bromide. For each gene, one amplification product was verified by DNA sequencing (Biofidal, Vaulx-en-Velin, France) after gel extraction (Qiagen, Hilden, Germany).

## Results

### *S. littoralis* Transcriptome and Annotation

**Figure 1:**
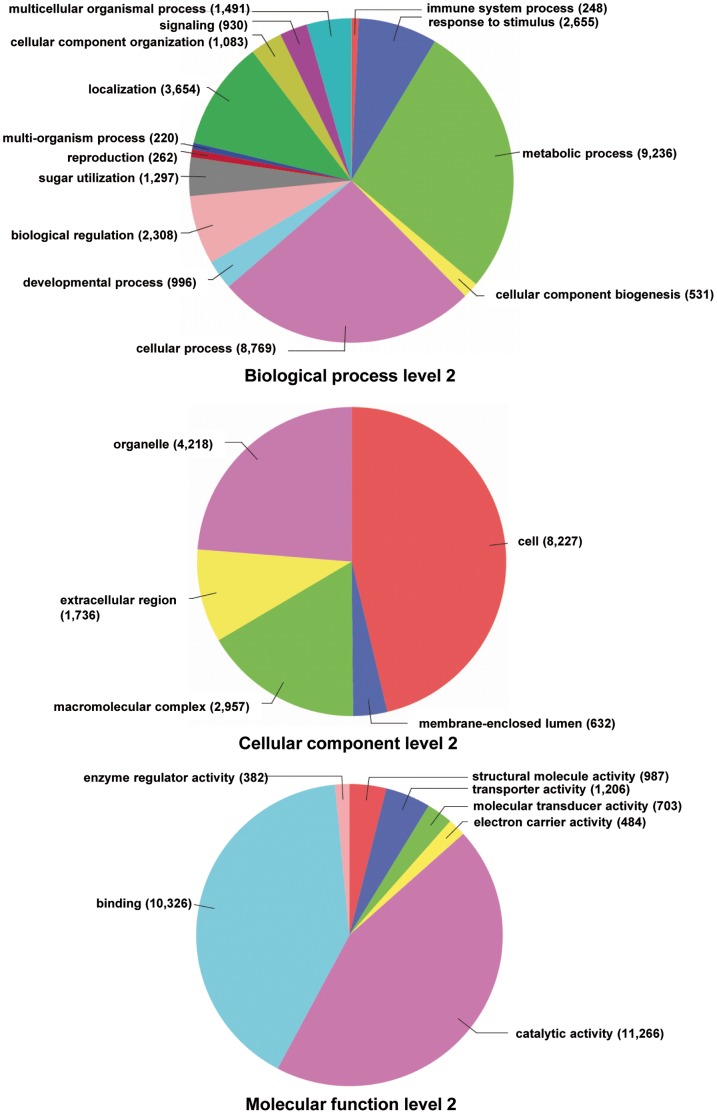
Distribution of *S. littoralis* contigs annotated at GO level 2.

We generated a *de novo* reference transcriptome of *S. littoralis*, by using several transcriptomic data sets obtained by Sanger, 454 and Illumina sequencing (Table 1). EST collections from male and female antennae, containing respectively 20,760 and 18,342 sequences, have been previously described [38], [43]. Raw 454 data sets were obtained from female antennae (656,772 sequences), male antennae (557,390 sequences) and various tissues (424,199 sequences). Raw Illumina data sets were obtained from fed (1,947,899 reads) and starved (2,389,809 reads) larvae chemosensory organs (see Materials and Methods). First, processed Illumina RNAseq (3,979,595 reads) served as input in Trinity to generate a first assembly into 11,560 contigs. Second, a data set containing 1,426,041 processed sequences including the 11,560 contigs generated previously by Trinity, together with Sanger EST and 454 data sets, was used as input in MIRA to generate a reference transcriptome assembly. The MIRA assembler provided as output a set of 77,643 contigs, ranging in length from 40 to 8,731 bp with a median size of 653 bp. It must be pointed out that these contigs do not represent unigenes, since their assembly took into account possible splice variants, polymorphism or reverse transcriptase errors. Among the 77,643 contigs, a coding region was predicted for 36,345 sequences (43.98%, mean length: 177 aa, median length: 154 aa, max length: 1907 aa, min length: 30 aa). All contigs were compared to the non-redundant protein database (NR, version 20.03.2012). Fig. 1 illustrates the distribution of the *S. littoralis* contigs in GO terms. Among the 77,643 *S. littoralis* contigs, 21,166 corresponded to at least one GO term. A large number of transcripts could not be associated with a GO term (72.7%). Among those associated to a GO-term, 18,765 were assigned to a molecular function (88.7%), 15,433 to a putative biological process (72.9%) and 9,920 to a cellular component (46.9%) (Fig. 1). In the molecular function category, the terms catalytic activity and binding were the most represented (44.4% and 40.7%, respectively). In the biological process category, the terms metabolic process and cellular process were the most represented (27.4% and 26%, respectively). In the cellular component category, the terms cell and organelle were the most represented (46.3% and 23.7%, respectively). In each of the three GO categories (level 2), the more abundant terms were the same as those observed in the adult antennae transcriptome [43].

### Identification of New Putative *S. littoralis* Olfactory Genes

**Figure 2:**
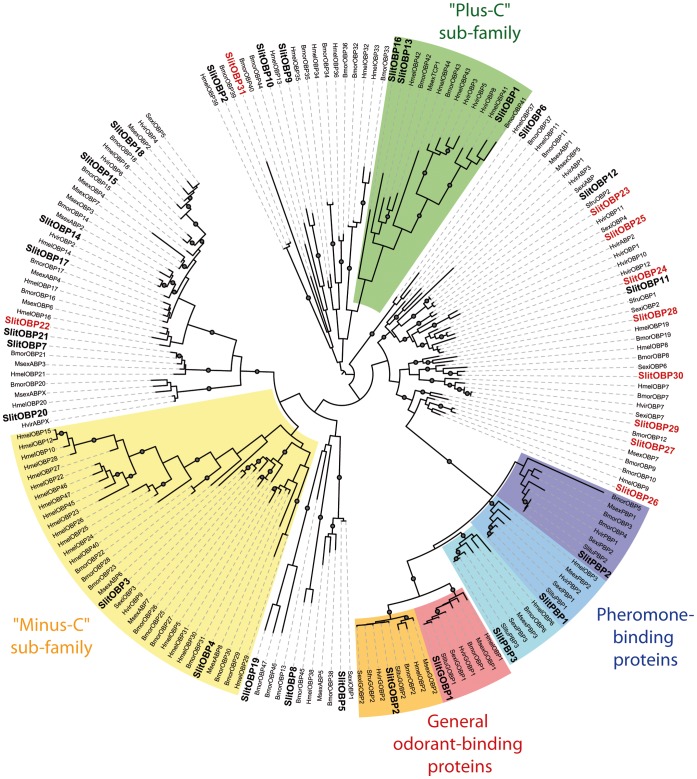
Maximum likelihood tree of candidate odorant-binding proteins (OBPs) from *S. littoralis* and other Lepidoptera. Sequences used were from *B. mori* [36], *H. melpomene* [41], *H. virescens* [56], [57], [58], *M. sexta* [37], [59] and from three other species of the genus *Spodoptera* (*S. exigua*, *S. frugiperda* and *S. litura*). Signal peptide sequences were removed from the data set. Branch support was estimated by approximate likelihood-ratio test (aLRT) (circles: >0.95) [70]. Images were created using the iTOL web server [89]. SlitOBPs are in bold and the new SlitOBPs identified in this study are in red.

A total of 56 and 68 sequences showing similarities with Lepidoptera *OBPs* and *CSPs*, respectively, were identified in the *S. littoralis* reference transcriptome. Assembly, when possible, and comparison with the 26 *OBPs* and 14 *CSPs* previously obtained revealed that 10 sequences were new *OBPs*, referred to as *SlitOBPs*, and that 7 sequences were new *CSPs*, referred to as *SlitCSPs* (Table 2). For convenience, *SlitOBPs* and *CSPs* were numbered according to their closest homologs whenever possible. This led to a total of 36 *OBPs* and 21 *CSPs* identified in *S. littoralis* antennae. Almost all the deduced proteins have the characteristic hallmarks of the OBP and CSP protein families: the presence of a signal peptide, and the highly conserved six (OBPs) and four (CSPs) cysteine profiles (Table 2). Some of the SlitOBPs clustered in the “plus-C” and “minus-C” OBP sub-families (Fig. 2), in correlation with their cysteine number. The SlitCSPs distributed in all groups of lepidopteran CSPs (Supporting Information S2). Some of the sequences were incomplete at their 5′ ends and the corresponding proteins missed the signal peptide (Table 2).

**Table 2:** List of *S. littoralis* contigs putatively involved in odorant binding.

Name	Signal peptide	Length (aa)	Blastx hit	e-value	Identity
SlitCSP15	Yes	122	|NP_001091781.1| chemosensory protein 15 [Bombyx mori]	2e−34	59%
SlitCSP16	Yes	129	|ACX53692.1| chemosensory protein [Heliothis virescens]	6e−32	51%
SlitCSP17	Yes	138	|BAG71921.1| chemosensory protein 13 [Papilio xuthus]	7e−66	77%
SlitCSP18	No	145	|EHJ73331.1| chemosensory protein 12 [Danaus plexippus]	4e−38	59%
SlitCSP19	Yes	123	|NP_001037067.1| chemosensory protein 8 precursor [Bombyx mori]	2e−53	64%
SlitCSP20	Yes	109	|AFR92094.1| chemosensory protein 10 [Helicoverpa armigera]	3e−65	87%
SlitCSP21	Yes	111	|NP_001037066.1| chemosensory protein precursor [Bombyx mori]	1e−43	63%
SlitOBP22	Yes	140	|EHJ65654.1| antennal binding protein 4 [Danaus plexippus]	1e−54	67%
SlitOBP23	Yes	145	|AAR28763.1| odorant-binding protein-2 precursor [Spodoptera frugiperda]	3e−70	82%
SlitOBP24	Yes	146	|AAR28762.1| odorant-binding protein [Spodoptera frugiperda]	1e−56	59%
SlitOBP25	Yes	147	|ADY17885.1| odorant binding protein [Spodoptera exigua]	3e−69	70%
SlitOBP26	Yes	154	|ADK47525.1| odorant binding protein [Manduca sexta]	1e−61	62%
SlitOBP27	Yes	153	|NP_001153664.1| odorant binding protein LOC100301496 precursor[Bombyx mori]	8e−53	56%
SlitOBP28	Yes	150	|NP_001140188.1| odorant-binding protein 4 [Bombyx mori]	4e−39	47%
SlitOBP29	No	129	|ADY17886.1| odorant binding protein [Spodoptera exigua]	2e−85	98%
SlitOBP30	No	170	|AFM77984.1| oderant binding protein 6 [Spodoptera exigua]	2e−74	71%
SlitOBP31	No	270	|EHJ73423.1| twelve cysteine protein 1 [Danaus plexippus]	8e−60	45%

Signal peptides were determined using SignalP 4.0 [52]. aa: amino acid.

**Figure 3:**
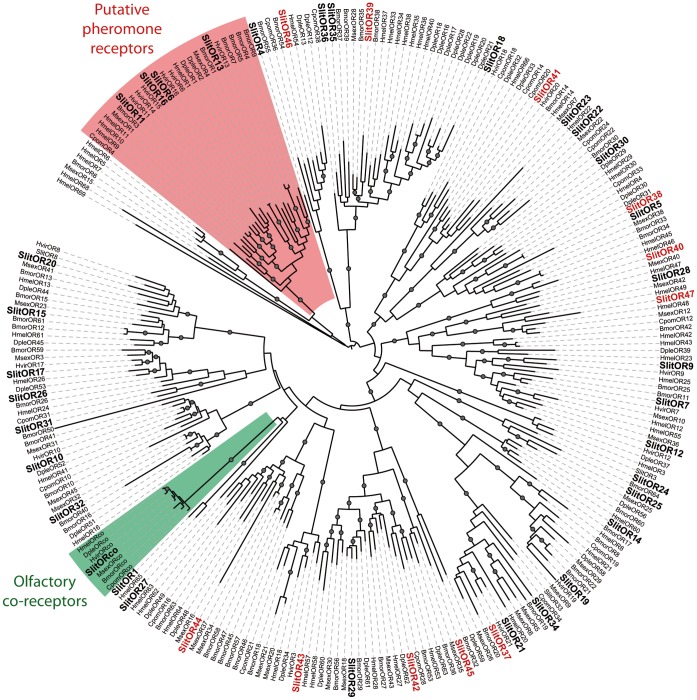
Maximum likelihood tree of candidate ORs from *S. littoralis* and other Lepidoptera. Sequences used were from *B. mori* [3], *C. pomonella* [39], *H. virescens* [54], [55], *M. sexta* [37], *D. plexippus* [40] and *H. melpomene* [41]. Branch support was estimated by approximate likelihood-ratio test (aLRT) (circles: >0.95) [70]. Images were created using the iTOL web server [89]. SlitORs are in bold and the new SlitORs identified in this study are in red.

A total of 11 new putative *OR* genes and 1 new gustatory receptor gene (*GR*) were identified in the *S. littoralis* reference transcriptome. Together with the 36 *ORs* and the 5 *GRs* previously annotated [38], [43], this led to a total of 47 *ORs* (referred to as *SlitORs*) and 6 *GRs* (referred to as *SlitGRs*) described in *S. littoralis*. For convenience, new *SlitORs* and *GRs* were numbered according to their closest homologs – when possible – from *H. virescens, M. sexta* or *B. mori* in the phylogenetic analyses (Fig. 3). Interestingly, we could correct the *SlitOR9* sequence that has been previously proposed to be a pseudogene [43]. Using RACE-PCR, we extended the sequences of *SlitOR46* and *SlitOR47*. SlitOR proteins shared between 41% and 83% identity with other lepidopteran ORs. Among the newly identified ORs, three sequences (SlitOR37, 41, 42) contained the seven transmembrane domains that characterize this family of proteins (Table 3). Depending of the size of the fragments, the other SlitORs exhibited between zero and six transmembrane domains (Table 3).

**Table 3:** List of *S. littoralis* contigs putatively involved in chemosensory reception.

Name	TM nb	Length (aa)	Blastp hit	e-value	Identity
SlitOR37	7	403	|CAG38122.1| putative chemosensory receptor 21 [Heliothis virescens]	6e−139	52%
SlitOR38	4	390	|NP_001103623.1| olfactory receptor 33 [Bombyx mori]	3e−69	32%
SlitOR39	2	111	|ABK27851.1| odorant receptor 38 [Bombyx mori]	7e−42	62%
SlitOR40	0	129	|EHJ76372.1| putative Odorant receptor 85d [Danaus plexippus]	4e−20	49%
SlitOR41	7	362	|ACC63240.1| olfactory receptor 20, partial [Helicoverpa armigera]	0	80%
SlitOR42	7	404	|NP_001166893.1| olfactory receptor 27 [Bombyx mori]	6e−117	54%
SlitOR43	6	373	|CAD31852.1| putative chemosensory receptor 3 [Heliothis virescens]	0	77%
SlitOR44	0	78	|AFC91724.1| putative odorant receptor OR16 [Cydia pomonella]	6e−09	68%
SlitOR45	4	366	|NP_001166892.1| NP_001166892.1 olfactory receptor 36 [Bombyx mori]	2e−146	60%
SlitOR46	6	366	|BAG12812.1| olfactory receptor 54 [Bombyx mori]	5e−156	63%
SlitOR47	2	144	|AFC91721.1| putative odorant receptor OR12 [Cydia pomonella]	5e−40	50%
SlitIR8a	1	162	|AFC91764.1| putative ionotropic receptor IR8a, partial [Cydia pomonella]	1e−50	79%
SlitIR2	0	133	|EHJ76709.1| ionotropic glutamate receptor-invertebrate[Danaus plexippus]	1e−32	61%
SlitIR3	1	284	||EHJ72198.1| putative ionotropic glutamate receptor-invertebrate[Danaus plexippus]	2e−30	30%
SlitIR4	2	146	|EHJ72198.1| putative ionotropic glutamate receptor-invertebrate[Danaus plexippus]	3e−26	43%
SlitIR64a	0	219	|EHJ70236.1| hypothetical protein KGM_00806 [Danaus plexippus]	2e−66	58%
SlitGR6	2	137	|CAD31947.1| putative chemosensory receptor 5 [Heliothis virescens]	8e−29	45%

Transmembrane domains (TM) were predicted using TMHMM version v.2.0 [53]. aa: amino acid.

**Figure 4:**
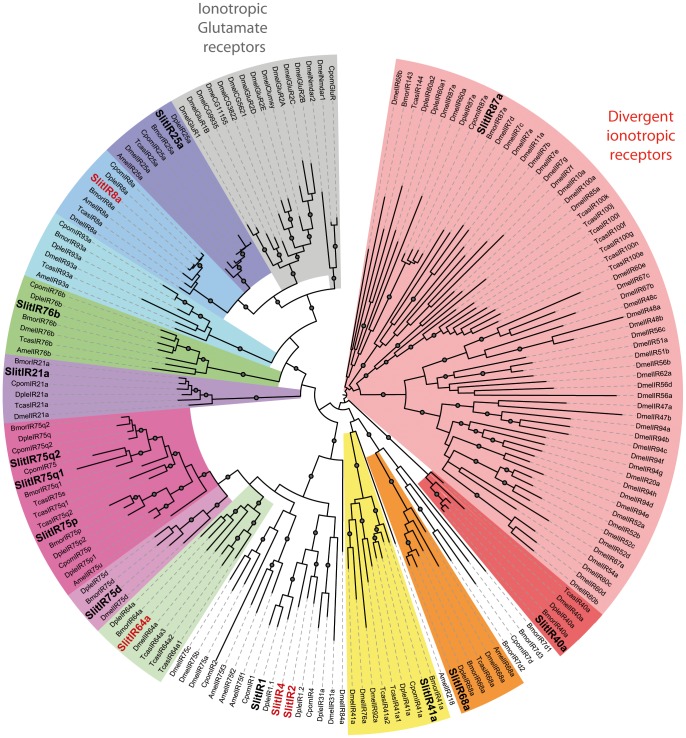
Maximum likelihood tree of candidate ionotropic receptors (IRs) from *S. littoralis* and other insects. Sequences used were from *B. mori* [11], *C. pomonella* [39], *D. plexippus* [40], *D. melanogaster*, *Apis mellifera* and *Tribolium castaneum* [11]. Branch support was estimated by approximate likelihood-ratio test (aLRT) (circles: >0.95) [70]. Images were created using the iTOL web server [89]. SlitIRs are in bold and the new SlitIRs identified in this study are in red.

We previously annotated twelve *IR* sequences in *S. littoralis* [63] and here we identified five new putative *SlitIRs* (Table 3), leading to a total of seventeen candidate *IRs*. A phylogenetic analysis conducted with the whole set of SlitIR proteins (except SlitIR3 that was too small to be included) and other insect IRs revealed that we found the *S. littoralis* member of the IR8 sub-family, suspected to be a co-receptor for other IRs [72], like IR25a (Fig. 4). We also identified a member of the IR64a subfamily, whose homolog in *D. melanogaster* is involved in acid sensing [12]. Interestingly, SlitIR1, SlitIR2 and SlitIR4 appeared in a group that included only lepidopteran IR proteins, supporting our previous hypothesis of the occurrence of a lepidopteran specific IR sub-goup [39], [63].

All the amino acid sequences deduced from the new chemosensory genes identified in this study are provided in Supporting Information S3.

### RT-PCR in Adults and Larvae

We retrieved all *S. littoralis* putative chemosensory gene transcripts (further referred to as *SlitOBPs, SlitCSPs, SlitPBPs, SlitGRs, SlitORs*, and *SlitIRs*) by PCR, except six genes for which we failed to amplify PCR products in spite of numerous tries in the four tissues. These genes consisted of *SlitCSP1, SlitCSP15, SlitCSP16, SlitIR1, SlitIR75d* and *SlitIR75q2*. Since these gene sequences derived from the assembly of reads, it is possible that they result from mis-assembly and/or contain sequence errors. Alternatively, since the reference transcriptome used to predict *S. littoralis* olfactory genes results from the assembly of sequences obtain from various tissues, including whole larvae, adult brain, and gut, it is possible that some of these genes are not expressed in chemosensory organs but in other tissues. However, *SlitIR1, SlitIR75d* and *SlitIR75q2* could be amplified in an earlier study [63] and we included thereafter the expression profile of these three genes in this study (Table 4).

**Table 4:** RT-PCR amplifications of *SlitOBP* (including *PBP* and *GOBP*), *CSP, GR, IR* and *OR* transcripts in adult male and female antennae (ant) and larvae antennae, and larvae maxillary palps (palp).

	Larvae		Adults			Larvae		Adults	
Name	ant	palp	♂ ant	♀ ant	Name	ant	palp	♂ ant	♀ ant
*SlitGOBP1*	**0**	**0**	**1**	**1**	*SlitIR1*	**0** 1	NA^1^	**1** 1	**1** 1
*SlitGOBP2*	**1**	**1**	**1**	**1**	*SlitIR21a*	**0**	**0**	**1**	**1**
*SlitOBP1*	**1**	**1**	**1**	**1**	*SlitIR25a*	**1**	**1**	**1**	**1**
*SlitOBP2*	**0**	**1**	**1**	**1**	*SlitIR40a*	**0**	**0**	**1**	**1**
*SlitOBP3*	**1**	**0**	**1**	**1**	*SlitIR41a*	**0**	**1**	**1**	**1**
*SlitOBP4*	**1**	**0**	**0**	**1**	*SlitIR68a*	**1**	**1**	**1**	**1**
*SlitOBP5*	**0**	**1**	**1**	**1**	*Slit75d*	**0** 1	NA^1^	**1** 1	**1** 1
*SlitOBP6*	**1**	**0**	**0**	**1**	*SlitIR75p*	**0**	**1**	**1**	**1**
*SlitOBP7*	**1**	**1**	**1**	**1**	*SlitIR75q1*	**0**	**0**	**1**	**1**
*SlitOBP8*	**1**	**1**	**1**	**1**	*SlitIR75q2*	**0** 1	NA^1^	**1** 1	**1** 1
*SlitOBP9*	**1**	**1**	**1**	**1**	*SlitIR76b*	**0**	**1**	**1**	**1**
*SlitOBP10*	**1**	**1**	**1**	**1**	*SlitIR87a*	**0**	**0**	**1**	**1**
*SlitOBP11*	**1**	**1**	**1**	**1**	*SlitIR8a*	**1**	**1**	**1**	**1**
*SlitOBP12*	**0**	**1**	**1**	**1**	*SlitIR3*	**0**	**1**	**1**	**1**
*SlitOBP13*	**1**	**1**	**1**	**1**	*SlitIR64a*	**1**	**1**	**1**	**1**
*SlitOBP14*	**1**	**1**	**1**	**1**	*SlitIR2*	**0**	**1**	**1**	**1**
*SlitOBP15*	**1**	**1**	**1**	**1**	*SlitIR4*	**0**	**1**	**1**	**1**
*SlitOBP16*	**1**	**1**	**1**	**1**	*SlitOR1*	**0**	**0**	**1**	**1**
*SlitOBP17*	**1**	**1**	**1**	**1**	*SlitOR2*	**1**	**1**	**1**	**1**
*SlitOBP18*	**1**	**1**	**1**	**1**	*SlitOR3*	**0**	**1**	**1**	**1**
*SlitOBP19*	**1**	**1**	**1**	**1**	*SlitOR4*	**0**	**0**	**1**	**1**
*SlitOBP20*	**1**	**1**	**1**	**1**	*SlitOR5*	**0**	**0**	**1**	**1**
*SlitOBP21*	**0**	**0**	**1**	**1**	*SlitOR6*	**0**	**0**	**1**	**0**
*SlitOBP22*	**1**	**1**	**1**	**1**	*SlitOR7*	**1**	**1**	**1**	**1**
*SlitOBP23*	**1**	**1**	**1**	**1**	*SlitOR8*	**1**	**1**	**1**	**1**
*SlitOBP24*	**1**	**1**	**1**	**1**	*SlitOR9*	**1**	**1**	**1**	**1**
*SlitOBP25*	**1**	**1**	**1**	**1**	*SlitOR10*	**0**	**0**	**1**	**1**
*SlitOBP26*	**1**	**1**	**1**	**1**	*SlitOR11*	**0**	**0**	**1**	**1**
*SlitOBP27*	**1**	**1**	**1**	**1**	*SlitOR12*	**0**	**0**	**1**	**1**
*SlitOBP28*	**1**	**1**	**1**	**0**	*SlitOR13*	**0**	**0**	**1**	**0**
*SlitOBP29*	**0**	**1**	**1**	**0**	*SlitOR14*	**1**	**0**	**1**	**1**
*SlitOBP30*	**0**	**1**	**0**	**0**	*SlitOR15*	**1**	**0**	**1**	**1**
*SlitOBP31*	**1**	**1**	**1**	**1**	*SlitOR16*	**0**	**0**	**1**	**1**
*SlitPBP1*	**1**	**0**	**1**	**1**	*SlitOR17*	**0**	**0**	**1**	**1**
*SlitPBP2*	**1**	**0**	**1**	**1**	*SlitOR18*	**1**	**1**	**1**	**1**
*SlitPBP3*	**1**	**0**	**1**	**1**	*SlitOR19*	**0**	**0**	**1**	**1**
*SlitCSP1*	NA	NA	NA	NA	*SlitOR20*	**0**	**0**	**1**	**1**
*SlitCSP2*	**1**	**1**	**1**	**1**	*SlitOR21*	**0**	**0**	**1**	**1**
*SlitCSP3*	**1**	**1**	**1**	**1**	*SlitOR22*	**1**	**1**	**1**	**1**
*SlitCSP4*	**1**	**1**	**1**	**1**	*SlitOR23*	**0**	**0**	**1**	**1**
*SlitCSP5*	**1**	**1**	**1**	**1**	*SlitOR24*	**1**	**1**	**1**	**1**
*SlitCSP6*	**0**	**1**	**1**	**0**	*SlitOR25*	**1**	**1**	**1**	**1**
*SlitCSP7*	**1**	**1**	**1**	**1**	*SlitOR26*	**0**	**0**	**1**	**1**
*SlitCSP8*	**1**	**1**	**1**	**1**	*SlitOR27*	**0**	**0**	**1**	**1**
*SlitCSP9*	**1**	**1**	**1**	**1**	*SlitOR28*	**0**	**0**	**1**	**1**
*SlitCSP10*	**1**	**1**	**1**	**1**	*SlitOR29*	**1**	**0**	**1**	**1**
*SlitCSP11*	**1**	**1**	**1**	**1**	*SlitOR30*	**0**	**0**	**1**	**1**
*SlitCSP12*	**1**	**1**	**1**	**1**	*SlitOR31*	**0**	**0**	**1**	**1**
*SlitCSP13*	**0**	**1**	**1**	**1**	*SlitOR32*	**1**	**0**	**1**	**1**
*SlitCSP14*	**1**	**1**	**1**	**1**	*SlitOR33*	**0**	**0**	**1**	**1**
*SlitCSP15*	NA	NA	NA	NA	*SlitOR34*	**0**	**0**	**1**	**1**
*SlitCSP16*	NA	NA	NA	NA	*SlitOR35*	**0**	**0**	**1**	**1**
*SlitCSP17*	**1**	**1**	**1**	**1**	*SlitOR36*	**1**	**1**	**1**	**1**
*SlitCSP18*	**1**	**1**	**1**	**1**	*SlitOR37*	**1**	**1**	**1**	**1**
*SlitCSP19*	**0**	**1**	**1**	**1**	*SlitOR38*	**1**	**1**	**1**	**1**
*SlitCSP20*	**1**	**1**	**1**	**1**	*SlitOR39*	**1**	**1**	**1**	**1**
*SlitCSP21*	**1**	**1**	**0**	**0**	*SlitOR40*	**0**	**0**	**1**	**1**
*SlitGR1*	**0**	**0**	**1**	**1**	*SlitOR41*	**0**	**0**	**1**	**1**
*SlitGR2*	**0**	**0**	**1**	**1**	*SlitOR42*	**0**	**0**	**1**	**1**
*SlitGR3*	**1**	**1**	**1**	**1**	*SlitOR43*	**1**	**1**	**1**	**1**
*SlitGR4*	**1**	**1**	**1** 2	**1** 2	*SlitOR44*	**1**	**1**	**1**	**1**
*SlitGR5*	**1**	**1**	**1**	**1**	*SlitOR45*	**1**	**0**	**1**	**1**
*SlitGR6*	**0**	**0**	**1**	**1**	*SlitOR46*	**1**	**0**	**1**	**1**
					*SlitOR47*	**1**	**1**	**1**	**1**

1: expression; 0: not detected; NA: data not available.

1 [63],

2 [38].

**Figure 5:**
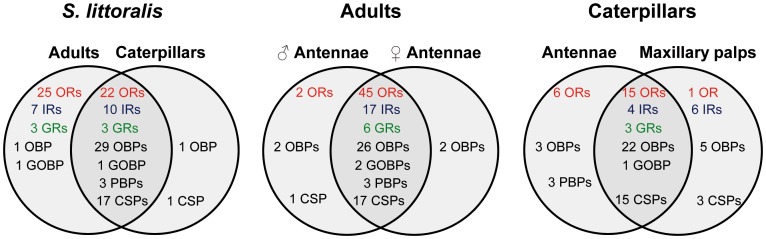
Distribution of chemosensory genes in *S. littoralis* adults and larvae. RT-PCRs were performed on male and female adult antennae and caterpillars antennae and maxillary palps. OBP: odorant-binding protein, PBP: pheromone-binding protein, GOBP: general odorant-binding protein, CSP: chemosensory protein, OR: olfactory receptor, IR: ionotropic receptor, GR: gustatory receptor.

Out of the 57 putative binding-proteins predicted in *S. littoralis* (33 OBPs, 3 PBPs and 21 CSPs), 51 transcripts were detected in both adult and larval chemosensory tissues, including the three *SlitPBPs*, as previously observed [73] (Fig. 5, Table 4). Two transcripts appeared to be adult-specific: *SlitGOBP1* (General odorant-binding protein 1, belonging to the *OBP* family) and *SlitOBP21,* and two genes were exclusively detected in larvae (*SlitOBP30* and *SlitCSP21*) (Fig. 5, Table 4). In adults, 48 *OBPs/CSPs* were expressed in antennae of both sexes, whereas two *OBPs* were female-specific (*SlitOBP4* and *SlitOBP6*) and three binding protein transcripts were male-specific (*SlitOBP28*, *SlitOBP29* and *SlitCSP6*) (Fig. 5, Table 4). In caterpillars, 38 *OBPs/CSPs* were detected in the two olfactory organs (the antennae and the maxillary palps), including *SlitCSP21* that was not found in adult antennae. Six *SlitOBPs* were antennae-specific (the 3 *SlitPBPs*, *SlitOBP3*, *SlitOBP6* and *SlitOBP4*) and 8 *OBPs/CSPs* were palp-specific (*SlitOBP2, 5, 12, 29, 30, SlitCSP6, 13 and 19*) (Fig. 5, Table 4), including *SlitOBP30* that was not found to be expressed in adult antennae (a representative gel picture is visible in Supporting Information S4).

We identified 47 *OR* genes in the *S. littoralis* transcriptome, including four putative *PR* genes (*SlitOR6, SlitOR11, SlitOR13*, and *SlitOR16*). Adults and larvae expressed a common *OR* repertoire of 22 genes, whereas 25 *ORs*, including the four putative *PRs*, appeared to be adult-specific (Fig. 5, Table 4). Male and female antennae expressed a common repertoire of 45 *ORs*, including two of the putative *PRs* (*SlitOR11* and *SlitOR16*). As previously reported [38], the two other putative *PRs* (*SlitOR6* and *SlitOR13*) were found to be male-specific. No female-specific *ORs* could be identified. In caterpillars, 15 *ORs* were found to be expressed in both antennae and palps, whereas one was palp-specific (*SlitOR3*) and six were antennae-specific (*SlitOR14, 15, 29*, *32, 45* and *48*) (Fig. 5, Table 4).

Out of the 17 *IRs* annotated in *S. littoralis*, 10 *IRs* were detected in both adults and larvae, including the two proposed *IR* co-receptor genes, *SlitIR8a* and *SlitIR25a* [72], whereas 7 *IRs* appeared to be adult-specific. In adults, the 17 *IRs* were found to be expressed in both male and female antennae. In caterpillars, 6 *IRs* were palp-specific (*SlitIR2, 3, 4, 41a, 75p* and *76b*) and no *IR* was found to be antennae-specific (Fig. 5, Table 4).

We also annotated six candidate *GRs* in *S. littoralis*. They do not represent the complete *GR* repertoire of this species, but as five were previously shown to be expressed in antennae [38], [43], they were investigated in this study. The six *GRs* were found to be expressed in both male and female antennae, whereas only three (*SlitGR3, 4, 5*) were expressed in caterpillars. These three *GRs* were expressed in both larval antennae and palps (Fig. 5, Table 4).

## Discussion

### A repertoire of Chemosensory Genes Identified in *S. littoralis*

We previously described members of the different olfactory gene families in *S. littoralis* by transcriptomic sequencing of adult antennae, establishing the use of such an approach to identify a large array of divergent *ORs* in a species with no genomic data available [38], [43]. Other lepidopteran species have been investigated the same way for description of *ORs*, such as *M. sexta* [37] and *C. pomonella* [39], but in all these studies only transcripts from adult antennae were sequenced. Here, we completed the *S. littoralis* transcriptomic data set by *de novo* sequencing larval tissues and re-sequencing adult antennae. The total number (36) of candidate *SlitOBPs* identified is a bit smaller than the 44 annotated *OBPs* found in the genome of *B. mori* [36] but higher than the 18 putative *OBPs* identified in the transcriptome of *M. Sexta* [37]. Eighteen putative *CSPs* have been annotated in *B. mori* [35] and 21 in *M. sexta* [37], this last number being identical to the 21 *CSPs* we identified in *S. littoralis*. These comparisons suggest that we have identified the nearly complete set of *S. littoralis OBPs/CSPs*, and confirm that Lepidoptera express a higher number of *CSPs* than other insect orders, such as Diptera [74]. We also annotated a large array of 47 candidate *SlitORs*, a number close to the numbers of *ORs* identified in other moths via similar transcriptomic strategies (*M. Sexta* : 47 *ORs* [37], *C. pomonella:* 43 *ORs* [39]). However, 66 *ORs* were annotated in the genome of *B. mori*, and 63 glomeruli were identified in the antennal lobe of *S. littoralis* [75]. Considering the one receptor-one glomerulus paradigm [76], [77], by which the number of expected *ORs* in a given species should correlate with the number of glomeruli in the antennal lobe, one would expect that there are still some *S. littoralis ORs* to identify.

### Adult and Larvae Express Similar Numbers of OBPs/CSPs in their Olfactory Organs

Only two studies, conducted in *B. mori*, investigated in detail the expression pattern of *OBPs* and *CSPs* [35], [36]. They revealed that most *OBPs* and *CSPs* are expressed throughout the insect development, including pupae. In a similar way, we found that most of these genes presented an overlapping expression between adults and larvae, between male and female antennae, and between larvae antennae and palps in *S. littoralis*. Some others presented restricted expression patterns, the biological signification of which is discussed below, although one has to keep in mind that the presence of a given RNA does not necessarily mean that the encoded protein is expressed. Since OBPs are proposed to participate in odor discrimination by binding a defined group of molecular structures [4], the OBP specifically expressed in caterpillars (*SlitOBP30*) may define larvae-specific olfactory capacities. CSPs were first defined as chemosensory proteins, but several expression studies revealed that they are expressed throughout the body [6], [78] and may participate in other physiological processes beyond chemoreception, such as development [79]. Thus, it is possible that the larvae-specific CSP (*SlitCSP21*), in addition to the other CSPs expressed in larvae, participates in larval development. The two adult-specific binding proteins (*SlitGOBP1, SlitOBP21*) may define adult-specific olfactory behaviors. Interestingly the *SlitGOBP2* gene was detected not only in adult antennae but also in caterpillar antennae and palps, contrary to *SlitGOBP1* which appeared to be adult specific. GOBPs constitute a monophyletic group of OBPs that are proposed to carry plant cues to the receptors since their expression is associated with plant volatile sensitive basiconic sensilla [80]. In a previous study conducted in *M. sexta*, *GOBP2* was also found to be expressed in larvae antennae, but *GOBP1* was not investigated [81]. These results suggest that, contrary to *GOBP2*, *GOBP1* participates in the detection of adult-only host plants. Finally, two *OBP* transcripts (*SlitOBP28* and *SlitOBP29*) appeared to be male specific and two others (*SlitOBP4* and *SlitOBP6*) were only detected in female antennae. This result is particularly interesting since, apart for *PRs*, no difference was observed between male and female *OR* repertoires. These *OBPs* might then support sex specific olfactory behaviours, such as oviposition site search in females or pheromone detection in males.

### Expression of Olfactory Genes Involved in Sex Pheromone Detection

We recently demonstrated that the sex pheromone signal is not only relevant for adults searching for a mate but also for caterpillars searching for food [73]. Indeed, we showed that *S. littoralis* larvae are more attracted to a food source containing the sex pheromone than to a food source without it. The sex pheromone induced electrical responses in larval olfactory sensilla and, accordingly, the three *SlitPBPs* were shown to be expressed in larvae antennae, but no *PRs* could be identified as expressed in larvae [73]. In the present study, we confirmed these previous observations. Thus, the question remains of which ORs would be responsible for pheromone detection in caterpillars. In *B. mori*, a larvae-specific OR responds to bombykol [3]. We expected in the present work to find larvae-specific *OR* transcripts, but we did not. It is possible that they remain to be identified. Alternatively, some of the 22 *ORs* expressed in both adults and larvae may be uncharacterized *PRs*. Currently, only one SlitOR has been functionally characterized as a PR [25]. Further characterization of the 22 ORs expressed in larvae would help in understanding the molecular mechanisms of pheromone detection in larvae.

### The Caterpillar OR and IR Repertoires are Smaller than the Adult Ones

We have previously studied the expression of a set of *ORs* [38], [43] and a set of *IRs* [63] in male and female antennae and the data obtained here confirmed their distribution. In addition, we report the comparative expression of these genes in the olfactory tissues of caterpillars. Also, we investigated the expression of 11 new *SlitORs* and 5 new *SlitIRs*. This study revealed that adult and larvae *OR* and *IR* repertoires are different and that the larvae express in their olfactory organs a smaller number of *ORs* and *IRs* (47 *ORs* in adults versus 22 in larvae; 17 *IRs* in adults versus 10 in larvae).

This situation is similar to what has been observed for *ORs* in other species. *Drosophila* larvae express 25 *ORs* versus around 60 in adults [1], [77], *Aedes aegypti* larvae express 24 *ORs* versus 83 [82], and *B. mori* caterpillars express 24 *ORs* versus 35 in adults [3]. In all these species, larvae-specific *ORs* were identified, but we did not evidence any larvae-specific *OR* in *S. littoralis*. It has to be noticed that we performed RT-PCR, which does not reflect relative abundance. Some *ORs* detected in the larval organs may be present at very low levels in the adult antennae, but well amplified by RT-PCR. Apart from PRs, only a few moth ORs have been functionally characterized to date. Interestingly, one of the SlitORs (SlitOR3) found to be expressed in both adults and larvae is homologous to the citral receptor from *Epiphyas postvitana* OR3 [83], and another one (SlitOR29) is homologous to the linalool/citral/acetate receptor from *B. mori* [3]. All the 17 *SlitIRs* were found to be expressed in adult antennae of both sexes. In larvae, the majority of the 10 expressed *SlitIRs* appeared to be palp-specific, with only four being also expressed in the antennae, including the two proposed IR co-receptor genes [72], *SlitIR8a* and *SlitIR25a*. It has been proposed that the ancestral chemosensory function of IRs is likely to be in the detection of water-soluble, non-volatile compounds and that antennal IRs gained olfactory function [11]. Since the maxillary palps are involved in both olfaction and taste, it is possible that some of the *SlitIRs* expressed in these organs have a gustatory function. Only a few functional data are available for insect IRs. Interestingly, one adult-specific SlitIR (SliIR84a) is homologous to the phenylacetaldehyde IR receptor characterized in *Drosophila* [10], and several adult-specific SlitIRs from the IR75 sub-family are homologous to *Drosophila* IRs responding to diverse aldehydes and acids. Both adults and larvae expressed SlitIRs homologous to the *Drosophila* phenylethyl amine receptor (IR76b) or the acetic acid receptor (IR64a) [12]. The detection of these compounds by *S. littoralis* larvae remains to be verified.

### Three Gustatory Receptors are Adult-specific

None of the *SlitGRs* examined appeared to be larvae-specific whereas three were only found in adult antennae. Interestingly, one of the adult-specific GRs (SlitGR2) is homologous to insect CO_2_ receptors [43]. Accordingly, three CO_2_ *GR* genes have been shown to be expressed at negligible levels in mosquito larvae [84]. Several moths employ CO_2_ gradients to evaluate floral quality [85], [86] but it is not known whether caterpillars are able to detect CO_2_ or not. Another adult-specific GR (SlitGR1) has been proposed to be involved in the detection of oviposition sites since it has been shown to be highly expressed in female ovipositors [43]. Its adult-specific expression suggests that females use different cues than larvae to detect an appropriate host. However, another SlitGR (SlitGR4), expressed in both adults and larvae, has also been proposed to be involved in the detection of oviposition sites [43]. Indeed, *SlitGR4* could be found in female ovipositors and it is homologous to the *Papilio xuthus* receptor to synephrine, an oviposition stimulant in this species [87]. Also, SlitGR4 is homologous to *D. melanogater* GR43a, which has recently been found to respond to fructose and which is expressed in the fly gut and brain [88]. These results suggest that females may use more complex cues than larvae to find the host. One of the SlitGRs expressed in both adult and larvae olfactory organs (SlitGR5) belongs to the sugar sensing receptor family [43]. Accordingly, both moth and caterpillars can detect sugar.

This study greatly improved the description of the *S. littoralis* chemosensory transcriptome. Investigation of the expression profiles of 127 olfactory genes in this species clearly shows that moths and caterpillars differ in the repertoires they express, according to their ecology and physiology, but that a variety of genes are used by both developmental stages. This work not only represents the first complete expression study of olfactory genes in a crop pest moth, but also described the olfactory molecular equipment of caterpillars, a developmental stage that represents a major source of agricultural loss.

## Supporting Information

Supporting Information S1 **Primer pairs, annealing temperature (T°), and amplification product sizes (PCR experiments).**
(DOCX)

Supporting Information S2 **Maximum likelihood tree of candidate chemosensory proteins (CSPs) from** ***S. littoralis*** **and other Lepidoptera.**
(TIF)

Supporting Information S3 **New chemosensory sequences identified in** ***S. littoralis*** **in Fasta format.**
(DOCX)

Supporting Information S4 **Representative RT-PCR amplifications of OBP/CSP transcripts showing differential expression between sexes or developmental stages.**
(DOCX)
