# Supplementary material for: A Comparison of the Olfactory Gene Repertoires of Adults and Larvae in the Noctuid Moth Spodoptera littoralis
Source: PLoS One. 2013 Apr 2;8(4):e60263. doi: 10.1371/journal.pone.0060263 (PMC3614943; doi:10.1371/journal.pone.0060263)
Supplement: Supporting Information S1 — Primer pairs, annealing temperature (T°), and amplification product sizes (PCR experiments). (DOCX) [file pone.0060263.s001.docx]

**Supporting information S1**

**Primer pairs, annealing temperature (T°), and amplification product sizes (PCR experiments).**

| **Name** | **Forward primer** | **Reverse primer** | **T°** | **Size** |
| --- | --- | --- | --- | --- |
| SlitGOBP1 | TTCCTGGGCCCTTATAATACCT | CTTTAACCTCTTGACCGACAGC | 60 | 401 |
| SlitGOBP2 | AGGAGTGCAGAGAAGAGTCAGG | CTAAGTGCAGCAGTCAATGGTC | 60 | 423 |
| SlitOBP1 | ATACAGGTGCCTGAACAATCCT | TAGGGGATCTGTGGAGAGTAGC | 60 | 417 |
| SlitOBP2 | GAAGAACCACGACAAGTTCTCC | GGAATTTAGGGTTTTGGGATTC | 60 | 393 |
| SlitOBP3 | GACAAGATGAAATCGTTTGTGG | GGCATTAGACCCCTTTTAGCTT | 60 | 416 |
| SlitOBP4 | CCGCACTGAATGTCTAACTGAG | ACATAGTTCCAGGCAGTTTGGT | 60 | 273 |
| SlitOBP5 | CTGACCAGGTTAACAGCATTGA | GTCTTCCATAGGCTTTCCATTG | 60 | 260 |
| SlitOBP6 | GTTATCGCCACTGCACATACAT | CCATATTTTTGGCTTCGGTATC | 60 | 498 |
| SlitOBP7 | CTGCCCCTCTTTGTCATTTTAG | CAGTTGTTCACGCCTCAGAATA | 60 | 425 |
| SlitOBP8 | ATAGATGTGAACGACTGCATGG | CGTTTCTGAAGTGAGTGACCAG | 60 | 475 |
| SlitOBP9 | GGGAACAAGAATAACAGGAACG | TCAATGGACTCCTGAAGGAAAT | 60 | 452 |
| SlitOBP10 | GAACCTGCAAGTAAGGAACGTC | GACGTCACAGATGATTGCACTT | 60 | 410 |
| SlitOBP11 | AGGATCAGGAGTCCAAAGTTGA | AGCTTCTCCATCGCTTACAGAC | 60 | 414 |
| SlitOBP12 | TATTGCATAATGTCGGTTGTGC | CTCTATCGCATCCTTTATCTCCA | 60 | 412 |
| SlitOBP13 | TGAAGATATGCAGAGCTGTGGT | GGTTCCATTTGGCGTGTATAGT | 60 | 405 |
| SlitOBP14 | GAATTCTGGGAAAATGCTGAAG | TTTATCCAAACCAGGAAGGATG | 60 | 467 |
| SlitOBP15 | TGTGTCTATAAAATGGGACAGACC | CTTCATTCAAACCCGATTTACG | 60 | 251 |
| SlitOBP16 | AGGCTCTCCTACCTCTTCCATT | GTATTTTTCACCCTCCTCCACA | 60 | 490 |
| SlitOBP17 | GTTATGGGATGACAAGACAGCA | CGTACATACATTTAGCGGTCCA | 60 | 342 |
| SlitOBP18 | AGAAAAACGTGCCAACCTAAGA | GCAAATATCTTTATGGGCATCC | 60 | 261 |
| SlitOBP19 | GGACTCAGTGTCGTTCATCAAA | GACCGTAGATTTGTCCTTCAGC | 60 | 478 |
| SlitOBP20 | CCCTCCTCCATTGTATTTTTCA | CTACCACTGCGTGAGAGAGATG | 60 | 437 |
| SlitOBP21 | CCCTCCTCCATTGTATTTTTCA | CTACCACTGCGTGAGAGAGATG | 60 | 437 |
| SlitOBP22 | GATTTGTTGGGGACGACTTG | GCTGGGTCCTTTTCATACAAAC | 60 | 455 |
| SlitOBP23 | CTGGAAACTGGACTGAAGAACC | TTTTCACTGCAGATTAGGAGCA | 60 | 488 |
| SlitOBP24 | GGAGTGCAGAACCAGTTAAACC | GGCGTAATCATCATCATTGCTA | 60 | 331 |
| SlitOBP25 | GGAGTGCAGAACCAGTTAAACC | TAGTACACCTCTCTCGCATCCA | 60 | 446 |
| SlitOBP26 | GAACACTTGGATTGTCCCAAAT | AGCGTTCTCTGTCATGCATTTA | 60 | 479 |
| SlitOBP27 | CGGTGCAGAATGTATCAAAGAA | AGGTCGAATCCGAGTTGTTCTA | 60 | 405 |
| SlitOBP28 | TGATCAAGAGATCAAAGCATGG | ACTGATCGTTCACATCCCTTTT | 60 | 328 |
| SlitOBP29 | GGAGCTTAAGATGGAGTTCACAA | ATAGCTGCTCGATCACAACCTT | 60 | 325 |
| SlitOBP30 | GATTCCGAGTCTAAACCAGTGC | CTCTGGATCCTCAGCCAAATAC | 60 | 462 |
| SlitOBP31 | ACAGGAGTCGAAGGTTTACCAA | GGTGTGGGATGTTTAAGTCCAT | 60 | 340 |
| SlitPBP1 | CGAGGTTGTATCGATGGTGATA | GAAGAAGTTGGACCTCATGGAC | 60 | 419 |
| SlitPBP2 | GAAGACCCTGAATGTAGCCAAG | CTCAACTTAGGGGACCACTTTG | 60 | 492 |
| SlitPBP3 | GAAGTACATCGCCTCAGGATTC | CGTCCTATATTTCCGTGAGGAC | 60 | 419 |
| SlitCSP1 | GTTGGTGGGCGTGTTATTATTT | CTAGCTTGGGATCCTTGTGAAG | 60 | 328 |
| SlitCSP2 | GGGATCGAGATTCACCTATCAG | CGGGATGATTTTTCTTCACTTC | 60 | 324 |
| SlitCSP3 | CCGTAGGTTCTTCAGCTGTTCT | CTTTATAGCATCCGGAAGGTTCT | 60 | 251 |
| SlitCSP4 | TCAACTGTCTGATGGATCTTGG | AGGAATACTAGCGGCATCCATA | 60 | 389 |
| SlitCSP5 | CGACAGATACGACAACGTCAAT | TGCTTCAGTTTTCTGAGTTCCA | 60 | 306 |
| SlitCSP6 | TAATCTCCCCGTTTTGACCTAA | CCATTGGTTTACGTCGGTATTT | 60 | 365 |
| SlitCSP7 | TGTTTCGATTCCTCTAGCGTCT | CTTAAACTATCCCGCGTATTGG | 60 | 367 |
| SlitCSP8 | CGGGAGAACAAACCTCTCATAC | GTGAGCTCGTTCCAGTACTCCT | 60 | 352 |
| SlitCSP9 | GTCGTTCGGTAGAACATGATTG | CAATTCTTTGGCATCAGGTGTA | 60 | 389 |
| SlitCSP10 | CTGTTCAGCACAATAGGTCTCG | TGACCAATCCTTCAAGTCATTC | 60 | 343 |
| SlitCSP11 | GGGAGATTGTGGTCCTTACAAA | TGACCAATCCTTCAAGTCATTC | 60 | 317 |
| SlitCSP12 | CGATGAAGTGCATCTACGTGTT | TTGGCCTCAAGAGTATGGAAGT | 60 | 358 |
| SlitCSP13 | CTCTCACCTGGCTGGACATATT | ATCACAGCCTTGATCACCTTTT | 60 | 308 |
| SlitCSP14 | GGACAACATCAACGTAGACGAG | GCGTTCCTGAACACAATTTACA | 60 | 382 |
| SlitCSP15 | GCAGTAATGAACGGTGCAAGTA | GAAATATCCCTTGAAAGCGTTG | 60 | 305 |
| SlitCSP16 | TAGTCTGCATCAATCCAGTTCG | CTTGTGGGCTTTTCTTCGAGTA | 60 | 339 |
| SlitCSP17 | CAAAGCTTAGGAGCATGTGATG | AATCAAATACAGCAGTCGCAGA | 60 | 485 |
| SlitCSP18 | ATAGCTGTCTTCGCCTTAGCTG | CACTGAAGGCCTTTTCGTATTC | 60 | 397 |
| SlitCSP19 | TGTACAAGGCTTCCAAAACAAG | TAATTTGTCACACACGCAACG | 60 | 360 |
| SlitCSP2 | GGGATCGAGATTCACCTATCAG | CGGGATGATTTTTCTTCACTTC | 60 | 324 |
| SlitCSP20 | AGGGTTCTGGTTGTACTGTCGT | GTCGTTCTTCTTGACGAGGTCT | 60 | 386 |
| SlitCSP21 | TACGGAGAAACAGAAGGCTCTC | TGTGGCCGTGTGTACGTATAAT | 60 | 401 |
| SlitIR1 | AGTGTCGTGTGATGTGCTGC | ACAGCGGCTTCAAGAATGTT | 60 | ≈500 |
| SlitIR21a | TGTGGTTTTTGGTGCAGAAG | GCATCATCGCCTTCCTTAAA | 60 | ≈500 |
| SlitIR25a | TTGCTCGACAGTGACGATTC | CATGGTTTGCGTTATCGTTG | 60 | ≈500 |
| SlitIR40a | ATTCTTGAAAACGGCACTGG | TGTCCAATACCAAGCAAGCA | 60 | ≈500 |
| SlitIR41a | CGTTGCGATTGAGAGCATAA | ACTTTGTCGACTGAGCCGTT | 60 | ≈500 |
| SlitIR68a | AAGTCGAATCGTTTCCCATTA | GGATTTGGAAATGGCACTGT | 60 | ≈500 |
| Slit75d | GGACCTCAACTGTCCCGATA | GTGCGACACCCCATAGAAGT | 60 | ≈500 |
| SlitIR75p | ATGATAACGGAGACGGCAAC | GTCGCATGATGGTATTCGTG | 60 | ≈500 |
| SlitIR75q1 | ACGCACCTTTAGAAGCGAAA | CTGCTTTTAGCAGGGGTGTC | 60 | ≈500 |
| SlitIR75q2 | ATCGGTGAAGACAACAATGG | AACGTCCAAAATGTTCAGGC | 60 | ≈500 |
| SlitIR76b | GGTGCTCTAGCTGGGATGAG | AGACGTGGAAGAGTCCGAGA | 60 | ≈500 |
| SlitIR87a | CGGGAGTGGTGTACCGTAAT | TAAGATCCGCCTCACTGCTT | 60 | ≈500 |
| SlitIR8a | GAATTTTCGCCACAGACTGG | CGGATGCTGATGAGTCTGAA | 60 | 220 |
| SlitIR3 | GCAAAGGAAATTGCACCCTA | CTCCCATTACCACAGCCAGT | 60 | 200 |
| SlitIR64a | TCACAAGAAGGTTGCGACAG | GGCAGACCCACTTCGTACAT | 60 | 250 |
| SlitIR2 | CGCCCTGATATCCAGAACAT | TGCCTTCACATTTGACTTCG | 60 | 200 |
| SlitIR4 | ACAGCTCGAAAAGCCACAAT | TTTCTTCTTTCGCACCTCGT | 60 | 270 |
| SlitOR1 | CCTATTTCAACCAGAAGACGATG | GCTGGAGAAGAGTGAAGAATGAA | 60 | 872 |
| SlitOR2 | CCTCTTCTTCACTCACACCATC | TAGCGGGCATCTGACTCC | 60 | 129 |
| SlitOR3 | GTATGGGATGCTGGTGAGAGAAG | AGTGGATTGAAGACCTGGATATGC | 58 | 163 |
| SlitOR4 | CAAAGGTTTGTCAGCTAAAATGG | ACATCTAAAGGCAACACGAAAAA | 60 | 894 |
| SlitOR5 | CAGACGAAGCAGACACCAATTC | TGGGTACGCTGGGATGAGAC | 58 | 118 |
| SlitOR6 | GAAGATGAATCTGTGCAGGG | ATGAACCGATTTGGCAGCCA | 62 | 179 |
| SlitOR7 | CCTTCCTATCGATGGCTCTG | CCCAGGTACCACTTGCAGTT | 60 | 115 |
| SlitOR8 | CTGATGCTCTGCCACTTCTAGTT | GCAAAATGATTTGGTTTCCATAA | 60 | 885 |
| SlitOR9 | GATTATCGTGG TGATAGCGAGAC | CCCGTCATGTACCAACAACTATT | 60 | 971 |
| SlitOR10 | CATGGACAAGATTAAGAAGATCCA | AACAGCGTAGAATCGCTACAAAC | 60 | 503 |
| SlitOR11 | GTGTGGCAGCAGCGTATC | AACTTGATGGCAACCTCTTCC | 62 | 210 |
| SlitOR12 | CGTGCAGTTCCAACTTCTGA | CGGAGCTGGAAACAAAGTTC | 62 | 200 |
| SlitOR13 | AGCTCGATACGGACCTCTCA | TCGATTTGCAGTGTCCATGT | 62 | 144 |
| SlitOR14 | CGTCATCACCCACAACCTCAC | CCCAATAGTCACCCAGCCAAAG | 58 | 196 |
| SlitOR15 | AACCATGCCGTCGGATCAAT | ACGTAGGAAGGCCGAAAACA | 60 | 1270 |
| SlitOR16 | GCCACTGACGATAATACTGAC | GACTCCAACATTAGCAACACC | 62 | 211 |
| SlitOR17 | GTAGCGATCGGTAACACAACAAT | CGAGCTCTCCACTGTTACTTCAT | 60 | 414 |
| SlitOR18 | GCTGGGACCTTGATGAGTATTG | CACGCATTGGACGCAGTTATAG | 58 | 150 |
| SlitOR19 | AAACGTGACTCCGTGAGCTT | CCGCCATCAACGTATTTTCT | 62 | 148 |
| SlitOR20 | CATTCACACAAACATCCGTCAC | ACCCAGCGTACTTGTATCAAAG | 53 | 156 |
| SlitOR21 | CCACGACAGGTCTGTGCTTA | TCTTGAACTGATGGCTGACG | 58 | 133 |
| SlitOR22 | TGGCAGTGAAAAGCCTAACTGT | ATTCGGTGCGTAGACGACAA | 60 | 1450 |
| SlitOR23 | ATTTTATCACGGCCTTATGACCT | GTAGCGAGCGTTGCATTATTAGT | 60 | 944 |
| SlitOR24 | CGCATCCGTTTATCGACTTT | CAAACCAGACCACAAGAGCA | 60 | 116 |
| SlitOR25 | AGCTTTCTGTTCCTGGCGTA | ATGATGGTAGACCGCACTCC | 62 | 186 |
| SlitOR26 | ATACAGCGGCTCGTTCAATC | GTGCCACATCACGTCCATAG | 60 | 94 |
| SlitOR27 | ACCAAATTGGCGTTTCTGTC | ATGGTACAGTTGGGGGTTGA | 60 | 80 |
| SlitOR28 | TGTAACTGGCGAGGGAAATCAC | GCTCTATATGGCTGCGGTTGG | 58 | 133 |
| SlitOR29 | CGTCATCACCCACAACCTCAC | CCCAATAGTCACCCAGCCAAAG | 58 | 196 |
| SlitOR30 | TGGCTCGTGTTCGGTTTCG | TTGGTTGTGGGTAGATCAGGTC | 53 | 127 |
| SlitOR31 | TCAAAATGGAAGATAATGTAGCA | TCCCCGCTTCTTTCTATCT | 60 | 1325 |
| SlitOR32 | TCTGAATAGGGCGAAGTTTGTAA | TGTGTAGGTCTTCACTCGTAGCA | 60 | 944 |
| SlitOR33 | CGCAGACATGAGATCAGGAA | CCCACCAGACCACAGAGACT | 62 | 133 |
| SlitOR34 | CGCAATATGGGTGTCTTCCT | CATGTTGCTCGATTCCCTTT | 62 | 178 |
| SlitOR35 | TGCGACCTGCCGACTATG | CTCCTCACGAACACGAACC | 53 | 179 |
| SlitOR36 | GTCTCCATACTCCTGAGGGTTCT | GCTGCAAAAATGTATTCTCCAAC | 60 | 904 |
| SlitOR37 | CGTAGGCCTGAAAAGAGTGC | CAGGGAGATGACCAGGATGT | 60 | 295 |
| SlitOR38 | CGATCGCAAGAAGTGTTTGA | ACTTGCAAACCCAAGACCAG | 60 | 250 |
| SlitOR39 | GACGTGATGAGGGGACCTAA | CACCAGCTGTCAGGGTCATA | 60 | 283 |
| SlitOR40 | TCATCGAGCTCTTGACATGG | GTTGTGCTCAGTACCGCAGA | 60 | 273 |
| SlitOR41 | TGCGTGATGACATTTCCAGT | GCCTTGCAGTACAGGTCCAT | 60 | 272 |
| SlitOR42 | TCATGATTTGTGCCAGTGCT | GCCCAGCTGTGAAAACAATAG | 60 | 250 |
| SlitOR43 | GTGAGCATAGTCGGCGTACA | TATACAGGGCCTGGCTGAAG | 60 | 283 |
| SlitOR44 | CGGGGGAGGTAGCTTAGAAC | AATTGGCACGGCACTAAGTC | 60 | 290 |
| SlitOR45 | TCGTTGGCTGGGATACTCTT | TGCTCGATACCAAAGTGGTG | 60 | 294 |
| SlitOR46 | TGATGTGTACAACAGCGTGAAG | ATCTTGGAGCTGCTTTGACG | 60 | 269 |
| SlitOR47 | CACCGAACAGGGAGATATGC | GACTGACGAAGCATGACGAA | 60 | 527 |
| SlitGR1 | CGACATTTACCGCGAATTTT | TTGGGACGAGCCTCAATTAC | 60 | 114 |
| SlitGR2 | GCCGGTGTCCAAGATACACT | CATGCTGATTGCCGAAGTAA | 62 | 168 |
| SlitGR3 | AGGTATTCAATCACGGCTTCC | TGTGTTCTGTTCGCTGGTC | 55 | 105 |
| SlitGR4 | AACTGAACTGGGTTGTATTGCAC | TGCATACGACACTTCATTCAAAC | 64 | 197 |
| SlitGR5 | GTTTGTGTTGCTGGTGATGG | TTCGAGGCTAGGATCAAGGA | 62 | 159 |
| SlitGR6 | CGCACGAGCGTTGATTAGT | CTACAGCGCATGGAATGGTA | 60 | 262 |
