# Supplementary figures and images for: A Comparison of the Olfactory Gene Repertoires of Adults and Larvae in the Noctuid Moth Spodoptera littoralis
Source: PLoS One. 2013 Apr 2;8(4):e60263. doi: 10.1371/journal.pone.0060263 (PMC3614943; doi:10.1371/journal.pone.0060263)

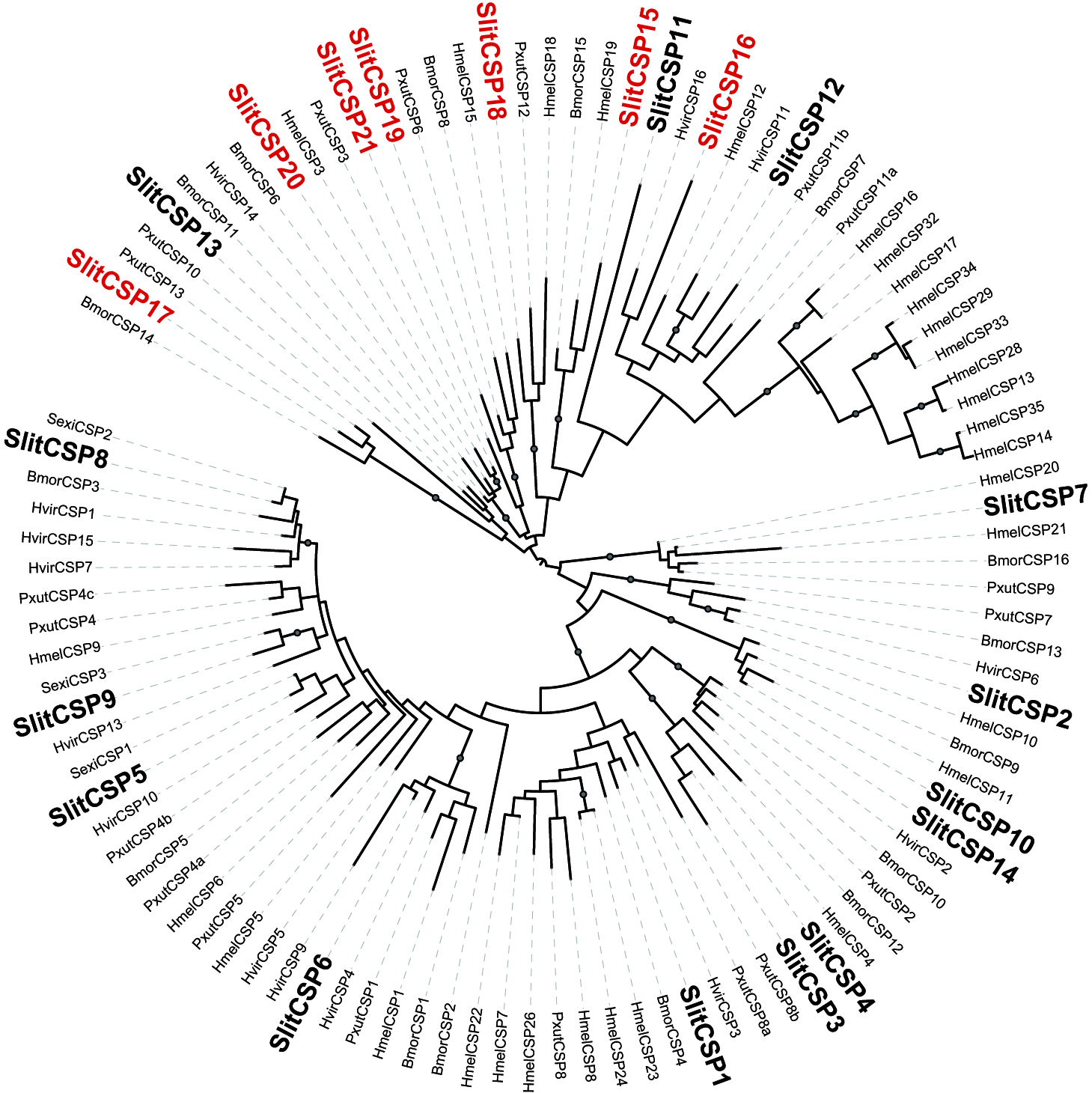

Supplement: Supporting Information S2 — Maximum likelihood tree of candidate chemosensory proteins (CSPs) from S. littoralis and other Lepidoptera. (TIF) [file pone.0060263.s002.tif]
