# Supplementary material for: A Comparison of the Olfactory Gene Repertoires of Adults and Larvae in the Noctuid Moth Spodoptera littoralis
Source: PLoS One. 2013 Apr 2;8(4):e60263. doi: 10.1371/journal.pone.0060263 (PMC3614943; doi:10.1371/journal.pone.0060263)
Supplement: Supporting Information S3 — New chemosensory sequences identified in S. littoralis in Fasta format. (DOCX) [file pone.0060263.s003.docx]

**Supporting information S3**

**New chemosensory sequences identified in *S. littoralis* in Fasta format**

>SlitOBP22

[M](http://web.expasy.org/cgi-bin/translate/dna_sequences?/work/expasy/tmp/http/seqdna.4432,3,170)NRLLLVYLVVLYAGCSYG[M](http://web.expasy.org/cgi-bin/translate/dna_sequences?/work/expasy/tmp/http/seqdna.4432,3,189)TRAQVKKT[M](http://web.expasy.org/cgi-bin/translate/dna_sequences?/work/expasy/tmp/http/seqdna.4432,3,198)GIIKNQC[M](http://web.expasy.org/cgi-bin/translate/dna_sequences?/work/expasy/tmp/http/seqdna.4432,3,206)PKNSVTEEQVGRIEQGVFIEDRNV[M](http://web.expasy.org/cgi-bin/translate/dna_sequences?/work/expasy/tmp/http/seqdna.4432,3,231)CYVACIYKSLQVVKNDKLD[M](http://web.expasy.org/cgi-bin/translate/dna_sequences?/work/expasy/tmp/http/seqdna.4432,3,251)ALITKQIDILYPPELKEPVKKSVAACFHSQDNYSDFCEGVFYASKCLYEKDPASFIFP

>SlitOBP26

[M](http://web.expasy.org/cgi-bin/translate/dna_sequences?/work/expasy/tmp/http/seqdna.4775,1,24)FNSSVF[M](http://web.expasy.org/cgi-bin/translate/dna_sequences?/work/expasy/tmp/http/seqdna.4775,1,31)YCLYFCALTPYLVSA[M](http://web.expasy.org/cgi-bin/translate/dna_sequences?/work/expasy/tmp/http/seqdna.4775,1,47)TAEQKALIHEHFETIGKSCNKDST[M](http://web.expasy.org/cgi-bin/translate/dna_sequences?/work/expasy/tmp/http/seqdna.4775,1,72)ITAEDIANLRAKKIPTGPNAPCFLAC[M](http://web.expasy.org/cgi-bin/translate/dna_sequences?/work/expasy/tmp/http/seqdna.4775,1,99)[M](http://web.expasy.org/cgi-bin/translate/dna_sequences?/work/expasy/tmp/http/seqdna.4775,1,100)KQIGV[M](http://web.expasy.org/cgi-bin/translate/dna_sequences?/work/expasy/tmp/http/seqdna.4775,1,106)DDNG[M](http://web.expasy.org/cgi-bin/translate/dna_sequences?/work/expasy/tmp/http/seqdna.4775,1,111)VQKETALE[M](http://web.expasy.org/cgi-bin/translate/dna_sequences?/work/expasy/tmp/http/seqdna.4775,1,120)AKAVFDDPEEIKAIEDYLHSCSHINTESVSDGAAGCERA[M](http://web.expasy.org/cgi-bin/translate/dna_sequences?/work/expasy/tmp/http/seqdna.4775,1,160)LAYKC[M](http://web.expasy.org/cgi-bin/translate/dna_sequences?/work/expasy/tmp/http/seqdna.4775,1,166)TENASKFGFDI

>SlitOBP31

RTEIDKSAYID[M](http://web.expasy.org/cgi-bin/translate/dna_sequences?/work/expasy/tmp/http/seqdna.7589,3,15)LANFTGSHQAWIRATEKVVSRCLNANNLERRSDCLINDVLACTLDVLTEYCPYKRKSGICKHGKHDVPCQISSSKSRPKNRREICLLPELVHHDHLYECDLDALYKVERVAVPVRHKKHVFPSLWHNCKQLETQTSCI[M](http://web.expasy.org/cgi-bin/translate/dna_sequences?/work/expasy/tmp/http/seqdna.7589,3,154)DK[M](http://web.expasy.org/cgi-bin/translate/dna_sequences?/work/expasy/tmp/http/seqdna.7589,3,157)GILNRYKF[M](http://web.expasy.org/cgi-bin/translate/dna_sequences?/work/expasy/tmp/http/seqdna.7589,3,166)DYFK[M](http://web.expasy.org/cgi-bin/translate/dna_sequences?/work/expasy/tmp/http/seqdna.7589,3,171)KDKIRQFTEDKPEWSA[M](http://web.expasy.org/cgi-bin/translate/dna_sequences?/work/expasy/tmp/http/seqdna.7589,3,188)[M](http://web.expasy.org/cgi-bin/translate/dna_sequences?/work/expasy/tmp/http/seqdna.7589,3,189)DIYTSAYINLP[M](http://web.expasy.org/cgi-bin/translate/dna_sequences?/work/expasy/tmp/http/seqdna.7589,3,201)YSDHCTSERKLLNVIDT[M](http://web.expasy.org/cgi-bin/translate/dna_sequences?/work/expasy/tmp/http/seqdna.7589,3,219)L[M](http://web.expasy.org/cgi-bin/translate/dna_sequences?/work/expasy/tmp/http/seqdna.7589,3,221)TCPVSKRKNTPQCNSLF[M](http://web.expasy.org/cgi-bin/translate/dna_sequences?/work/expasy/tmp/http/seqdna.7589,3,239)E[M](http://web.expasy.org/cgi-bin/translate/dna_sequences?/work/expasy/tmp/http/seqdna.7589,3,241)VKATPADNQTVTKEKAXXNYEALPPRXXVSDR

>SlitOBP25

[M](http://web.expasy.org/cgi-bin/translate/dna_sequences?/work/expasy/tmp/http/seqdna.19415,2,23)SKLSCIVFCAVA[M](http://web.expasy.org/cgi-bin/translate/dna_sequences?/work/expasy/tmp/http/seqdna.19415,2,36)RLCVFVASEDANSIFHAPIKPIVIDCAKEYGLSEDDIKKNRGLDGLKNLPPCFIRCVLNKLDIINDKGQYDADSGIATIKGL[M](http://web.expasy.org/cgi-bin/translate/dna_sequences?/work/expasy/tmp/http/seqdna.19415,2,119)SNNEYL[E](http://web.expasy.org/cgi-bin/translate/dna_sequences?/work/expasy/tmp/http/seqdna.19415,3,126)KISGVLKECESVNEKSVSDGDAGCERALLGA[M](http://web.expasy.org/cgi-bin/translate/dna_sequences?/work/expasy/tmp/http/seqdna.19415,3,158)CYLDHKTIVLA

>SlitOBP24

[M](http://web.expasy.org/cgi-bin/translate/dna_sequences?/work/expasy/tmp/http/seqdna.20302,1,29)YKIISLVFCIAVCLNRVHGNAEDKIAI[M](http://web.expasy.org/cgi-bin/translate/dna_sequences?/work/expasy/tmp/http/seqdna.20302,1,57)TAVKPFVEECAKKHGVTFEALLTAKASGKIDGVEPCFYSCVYKKTEFLNSKGEYDVDTALAKLKKYISNDDDYAKLSQVGKRCASVNSKPVGDGEAGCERGVLLTQCFLDHKGSVP[M](http://web.expasy.org/cgi-bin/translate/dna_sequences?/work/expasy/tmp/http/seqdna.20302,2,174)

>SlitOBP28

[M](http://web.expasy.org/cgi-bin/translate/dna_sequences?/work/expasy/tmp/http/seqdna.6946,2,14)IVRFLLCLYIVEFYGVHVQARTDQEIKAWFFREG[M](http://web.expasy.org/cgi-bin/translate/dna_sequences?/work/expasy/tmp/http/seqdna.6946,2,49)DCNIEHPISXKE[M](http://web.expasy.org/cgi-bin/translate/dna_sequences?/work/expasy/tmp/http/seqdna.6946,2,62)LELKENKIPDTNNAKCFVACVFKKTG[M](http://web.expasy.org/cgi-bin/translate/dna_sequences?/work/expasy/tmp/http/seqdna.6946,2,89)LDSXG[M](http://web.expasy.org/cgi-bin/translate/dna_sequences?/work/expasy/tmp/http/seqdna.6946,2,95)FDAENSIA[M](http://web.expasy.org/cgi-bin/translate/dna_sequences?/work/expasy/tmp/http/seqdna.6946,2,104)TQKDFANDPNRLESSKKLLETCKKVNDETVSDGEKGCERSVLLHKCFVETAPQLGIKLP

>SlitOBP29

[G](http://web.expasy.org/cgi-bin/translate/dna_sequences?/work/expasy/tmp/http/seqdna.7324,2,26)LTEEELK[M](http://web.expasy.org/cgi-bin/translate/dna_sequences?/work/expasy/tmp/http/seqdna.7324,2,34)EFTKLI[M](http://web.expasy.org/cgi-bin/translate/dna_sequences?/work/expasy/tmp/http/seqdna.7324,2,41)KCNKDGEVD[M](http://web.expasy.org/cgi-bin/translate/dna_sequences?/work/expasy/tmp/http/seqdna.7324,2,51)TELVQLQNYVVPTKQSTKCVLACAYKAAEV[M](http://web.expasy.org/cgi-bin/translate/dna_sequences?/work/expasy/tmp/http/seqdna.7324,2,82)NAKGEYDIDHAYKVAE[M](http://web.expasy.org/cgi-bin/translate/dna_sequences?/work/expasy/tmp/http/seqdna.7324,2,99)[M](http://web.expasy.org/cgi-bin/translate/dna_sequences?/work/expasy/tmp/http/seqdna.7324,2,100)KNGDEKRLVNAKK[M](http://web.expasy.org/cgi-bin/translate/dna_sequences?/work/expasy/tmp/http/seqdna.7324,2,114)ADLCVKVNELSVSDGEKGCDRAA[M](http://web.expasy.org/cgi-bin/translate/dna_sequences?/work/expasy/tmp/http/seqdna.7324,2,138)IFKCTVENAPKFGFKL

>SlitOBP30

HKFSAIQIPYNVLSRDDSNYLFVNNFNVVITKNYYETNNSKQALS[M](http://web.expasy.org/cgi-bin/translate/dna_sequences?/work/expasy/tmp/http/seqdna.7021,3,78)DDLKQKYVDNILECSKQYPIDRADAEQLQNRI[M](http://web.expasy.org/cgi-bin/translate/dna_sequences?/work/expasy/tmp/http/seqdna.7021,3,111)PDKEPIKCLFACVYKLAG[M](http://web.expasy.org/cgi-bin/translate/dna_sequences?/work/expasy/tmp/http/seqdna.7021,3,130)[M](http://web.expasy.org/cgi-bin/translate/dna_sequences?/work/expasy/tmp/http/seqdna.7021,3,131)NDQGELSVEGVNAISRKYLAEDPEKLQKSEEFTEACRSVNDAPVSDGTRGCDRAALIFKCTIEKSPEFNFV

>SlitOBP27

[Q](http://web.expasy.org/cgi-bin/translate/dna_sequences?/work/expasy/tmp/http/seqdna.5233,2,30)LFNSGVVFCIIVAVFLKNASAITDELKAHIEAKFLTVGAECIKEHPLTIEDLSAFKNRVFPDGENAGCFSACIFNKLGLFDDKGTLSHLTALENAKKVFEDQGELESIEKFLTTCAKVNDEEVSDGEKGCERAKLAYNCFIPKTIEQLGFDL

>SlitOBP23

[M](http://web.expasy.org/cgi-bin/translate/dna_sequences?/work/expasy/tmp/http/seqdna.5050,3,20)AKLSCLVLCVVAASLGSIHVAKGESLRESLRPVIVACSKEHGVTDAEIQAAKEAGSPASIKPCFIACVFKKAGFINEQGQLDLETGLKNLRQFVKDDEQYKQLEGVANKCSPVKDKAVSDGAAGCDRGVHLAGCFLDHKTSIII

>SlitCSP15

[I](http://web.expasy.org/cgi-bin/translate/dna_sequences?/work/expasy/tmp/http/seqdna.27225,3,26)AV[M](http://web.expasy.org/cgi-bin/translate/dna_sequences?/work/expasy/tmp/http/seqdna.27225,3,29)NGASKCNLLIF[M](http://web.expasy.org/cgi-bin/translate/dna_sequences?/work/expasy/tmp/http/seqdna.27225,3,41)FLFIATVVSQEKFYDRRYDYYEIDTLIQNPRLLKKYLDCFLWKGPCTPIGRVFRQILPEAVQTACKKCTPSQRRLARKXFNAFKGYFPEIHEELRKKLDP[K](http://web.expasy.org/cgi-bin/translate/dna_sequences?/work/expasy/tmp/http/seqdna.27225,2,170)KSTVK

>SlitCSP17

[T](http://web.expasy.org/cgi-bin/translate/dna_sequences?/work/expasy/tmp/http/seqdna.28813,2,15)PQEEHLHNFLSVTIN[M](http://web.expasy.org/cgi-bin/translate/dna_sequences?/work/expasy/tmp/http/seqdna.28813,2,31)RSWLLCLCVLTVVVSCYSQANRYENFNPDAIVQNDRILLAYYKCV[M](http://web.expasy.org/cgi-bin/translate/dna_sequences?/work/expasy/tmp/http/seqdna.28813,2,77)DKGPCTRDGKNFKRVLPETLATACGRCNPKQKTIVRKLLLGIRSKSEPRFLELLDKYNPDRSNRDALYAFLVTGA

>SlitCSP18

[S](http://web.expasy.org/cgi-bin/translate/dna_sequences?/work/expasy/tmp/http/seqdna.29237,6,1)TQSTRGWKPLGHRIKRIAIKFK[M](http://web.expasy.org/cgi-bin/translate/dna_sequences?/work/expasy/tmp/http/seqdna.29237,6,24)NALLIAVFALAAPLAFGYDEKYDKLDVDKILGDDALFTAYINC[M](http://web.expasy.org/cgi-bin/translate/dna_sequences?/work/expasy/tmp/http/seqdna.29237,6,68)LDKGPCSVEHSADFRQLLPEVIATACEKCTPIQRQNVRKTVKALSEKKPDDFVQFRAKFDPKGEYEKAFSAFVIGTD

>SlitCSP19

[M](http://web.expasy.org/cgi-bin/translate/dna_sequences?/work/expasy/tmp/http/seqdna.29504,5,13)KSILILCLLVAAVSCRPESYDTRFDNFDVEALVGNVRLLTAYGHCFLGNGPCTPEGSDFKKTIPDALRTGCGKCSPKQRHLIRVVVQGFQNKTPGLWQDLVKKQDPNGQYKEIFTRFLNGRD

>SlitCSP20

[M](http://web.expasy.org/cgi-bin/translate/dna_sequences?/work/expasy/tmp/http/seqdna.32027,1,158)RVLVVLSCLVVVAFAADKYNPKYDNFDVETLISNDRLLKAYINCFLEKGRCTPEGSDFKKALPEAIETTCAKCTDKQKGNIRKVIKAIQQKHPQEWEDLVKKNDPSGQ

>SlitCSP16

[M](http://web.expasy.org/cgi-bin/translate/dna_sequences?/work/expasy/tmp/http/seqdna.9040,2,50)KYILVALVVTIAVVKAQETYGTEYDNVNGEAIVSDDKQFQGFVDCFTGAAPCNEPAAAFKRVLPEAIVQACGKCNPAQKHLVRLFLEAYSKKSPQEYEKFKDLLILKGILSQIRSIRCRVLNYEIRSE

>SlitCSP21

[M](http://web.expasy.org/cgi-bin/translate/dna_sequences?/work/expasy/tmp/http/seqdna.13215,6,9)KLVIILALVAVXXARPDDGGFYDTKYDNFNADELIENERLLKSYAHCFLGDGKCTPEGNDFKKWIPEATTTSCGKCTEKQKALIAKTIKAIKDKLPSEYEALIKKHDPRK

>SlitOR37

METTTTSYTRSKTTNFFYKINFIVYIFGLPNFWIQDLKLSKRFSNKFYDKFSMFNNTLIFLLIIFELCSYFTQTQLTETHQSNRLIYAISHPMLFMFRVMMTSIKDRVKLVMYSLNVGLKRVHNDPEVEKQMIACTIMYLSALLLSCLMSMLMYAAEGFGEVIRHGKTFTTIITAYPRVEDASDMANVVRAICFIIWWIFLTRIFAVYILVISLTTCLSYQYKKPAELFHSLNDIFERNDLSQIEKEEQYEAGFKVGIKLHADTLRCTQLTQSVCRGVFSGQIVFNILLLVVLMAQMANSERTLVNLGSAGFTACAVLISTGFYMWNAGDVTVEASHLGTAIYFSGWYHCQGLSSVRIRKLVVITMSEAQRPVVLKGLGYIDLSYQSYIRIVKSSYSVFSVLF

>SlitOR38

MADQFEKTLKLFLVPMRLTLTSPEIPITFKWLIRYLLGFLTLTALAIVITYNTYISITNRVFFEACRNITLSLTYYGCCMNNILSFWHRSSLRALLETIRHDYKMAAQLPREEQLIFEDYDRKKCLMCKIWFHLFTVSLFLFVAKAVVLMIYYYFIGEFRLVHLYELTYPDFIEKRKGELVIYLFIFCIVFLYGLLSGLGFASFVPYGTICMLHACGHLEIARKRIDSLFTGDRRYVNEKLKNIAQLLQYTYKFMENTNGCFRLFYDATLKLSAIAIPFTLYALLEGLRHGEFSLEFTMFVLNAIXADQYTLLPKXXIIGKGEEVRLALYSCGWECEYNRRVRINILLLLTCCSRPIAMQTMFTTLCLYAVTDMFQQAYTILNLMNAVWN

>SlitOR39

REIEDVMRGPNAXGFLFLIVGLVAETXLGGLNNTILQLPFTLSQMGTDCFLGQKIMDANIKFEHAVYDCKWENFNQVNKKIVLVMLQNSQKTMTLTAGGMATLSFSYFILL

>SlitOR40

IIFVNLSSASISMCFFGFCAKVAHRALDMANNFVAVLALTLPLFNLCDYAERLREASAGMADSVYNNLWYISDKHYQKSXCGFILRRSQKPCCLTSLKYSEIGLNTFSAVLSTTWSYFSLASSLYESET

>SlitOR41

EMWRLASIPFLIATITPLATMIFIDMYKCWMAKDIVNIIRHSTVVGPFLGGFFKMILMYHKRIQAKQILDEIDRDYENINNYSEIYKDIARASVKNCQIYSERGWAITVVTCVMTFPVMAISLNVLQISCFKSEPVKYMIHDLEKPFSEDPEDRFESPYFEIIFVYMFYCSILYVVNFTGYDGFFGLAINXACLKMDLYCKALEEASKLDAHEVCGRVIGVIKEQCRMFQFVDLIQDTFNIWLGIIFLATMIQICTCLYHINEGYGFDLRYMIFVTGAVIHIYLPCRYAAKLKAMSLETANRFXSSGWEQVDDQRVRKMILFMVARAQVPNEIVALNMLAFNMELFVSILQTSYSMFTLLRS

>SlitOR42

KWYFKFLTVSEDPDNPIIGNYYLIASMWWSLGERSFXKLFSHNLVHFVAFIFVLSQYVELWVIRNDLEMAMRNLSLTMLSTVCVFKACNLVFWQNTWKELFDYVSELERSQLAKKDDTINKIIFQYVKYARRVTYLYWSLVTATVLIVSLAPLLIYWSSPTYRHNIRNGTVPYPEIMSSWTPFDRTRGIGFCVATVYXXXXXXXXXXXXXXXXXXXXXIMTFFAGQLKVLSANCSRLFGDGNELINCDETVKRIKECHLHHLYLIKFSAVLNSLLSPVMFLYVIICSXLMICASAAQLTTEGTTTVHQVWIAEYLMALIAQLFLYCWHSNDVFYLSNQVDDGVYSSAWWSQNVRIRRSLLLLGGQLRKPIVFTAGPFTKLNMATFVTILKGSYSYYTLVAKKED

>SlitOR43

NVFILIDDGFFSFNIKYLFFVGLWPEKTLTRNQKFLYKIYELFIHAITIIFLIMAGVGPIKNKDDILIVLSNLDKSLVVYNFFFKTIIFLIKRDQLSDLIDEIEASGDEVTKERKKLMANYVMAVTGMTAAVVSTFSLLALLEGIMSIEAWMPFDPVKNSMNLVLSLQIIAFCVFPGLCRAFAMQGLVCSMIMYSCDQLIHLQKELRSLDYVKDTEMITRMKFKMIIKKHIRLMGYSMKMESIFNEYFLVQNLAVTVELCLNAVMVSIVGVQQITLLFTFLAYLVLALINAYVYCYLGNELIIQSEGIALAAYESTWTSWPVDLQKDLLILINASQRPLKLSAGGIALLSIQTFSQALYNGYSIFAVLNDVVN

>SlitOR44

YCYQGNQLSEESFEIAGAAYECLWYKFSLRLRRALLIVMVXHXPSSTTHCWRIHYTFTCFFHGNHQSFVFIIHSATTS

>SlitOR45

QFQYFRVYRSFQKCRTNVRLGNLLKFLEDPEYPSVGPHLKLLGFTGLWHPNRQTLIGRFKQILFYVTITFFFSQYVKCFIKFNADSLKLILQYAPFHMGIVKSCFFQKDYKTWELVIDYMSSVEKKQLDKKDKKHDDIIHEYIKRNRKVSYFFWALAFFSNFSIFTEPYQKNQVNVNGTSIYLKIFDGYTPFDNEPPGYYFSMLIQTVLGHIVSAYVVGWDTLVVSIMIFFTGQLKITCMYCRLAIDALNSTKSHENIAKCHRFYTTLVEYTHLFNALVSPVMFMYLVVIAINLGVCIVQIVEIHDDITTLVSSILFVIACLIQLLLFYWFANEVTVESTFVSYSTFESDWPEANKKLQKEVALLG

>SlitOR46

GKQWYHAEDIESIVPKISGFSYRKDKVSAWFWYIHMFLLFYVYAVGNFWYQWKFAHGAGDFIKSYVNISIIVIIGNNSVWFVKQRPLLRTVLKKIEQSDELSRRSSFLMRKHEKLMKIVKRIVLIFYGFNYIDAFFIYFPHRVDLRNNYSMTPCVGLQPLTASPNREICMTILTLQEFTINIVALNYQALLLFLIAHTAAMYQMMAYEMMALNDYKKENLRQVKKKLSSLIERHCLTLDVVDNLRSLYSVPLGVNFGSNAVCISLFYLPLRECLQFMPVFVYCSLVFFLYCFLCQRLINSAEVFARAVYCCGWENFGLKEKRLVFVMLRQSQKPVELLAADIXPVNIYTFATTLQAMFKFVTVVKF

>SlitOR47

KLLNTSATYQVHQRFGGCFSVVNLINVLLSSVNICCVMFTIVFLDSWMEMSNKFYLGAALTQIFIVCWYADDIYRASVGVADAVYESGWYKSNTEVQARARRNFKRSQRPLYFTALKFRAITMITYSSILTTAYSYFTLLYTRD

>SlitGR6

FSSLFSVASIEHILSMISATGVGFPIGEFVYRYVTISHGFLLRAQDYSAWNGIPIFILSKLATVLWNFQDLIIILISMGLSSRYKRLNLYVRKIVTVEKRSETKPKFGTELYLQIQVWRRLREAYVRLINARAHGRP

>SlitIR8a

[Y](http://web.expasy.org/cgi-bin/translate/dna_sequences?/work/expasy/tmp/http/seqdna.28317,5,1)AIAVQQGSRLQEDLSRAXLELQKERFLEQLASKYWNESARQACPDADESEGITLESLGGVFIATLFGLGLA[M](http://web.expasy.org/cgi-bin/translate/dna_sequences?/work/expasy/tmp/http/seqdna.28317,5,73)LTLAWEVFYYKRKEKNKVQTFNSKPEKVAFEPKSSLESKLAQSVAKIRKRGKLGTRGNVAKNVTFGDSFKPVSEKGVSYISVFPKDYRP

>SlitIR3

HIKQLHNIK[M](http://web.expasy.org/cgi-bin/translate/dna_sequences?/work/expasy/tmp/http/seqdna.29744,3,13)LCQAVLTSILVHVIVGLSQDALQFGIEYFQYRGLKFICV[M](http://web.expasy.org/cgi-bin/translate/dna_sequences?/work/expasy/tmp/http/seqdna.29744,3,53)TCDKDFSWTVQYSKASQNLS[M](http://web.expasy.org/cgi-bin/translate/dna_sequences?/work/expasy/tmp/http/seqdna.29744,3,74)AISGVSIDSSVLDYNRVENCLQRKLHPIGVIVDADCGSTEDV[M](http://web.expasy.org/cgi-bin/translate/dna_sequences?/work/expasy/tmp/http/seqdna.29744,3,117)NFASQNIIWLDSNHKWLLIDDDEAYTWCNDTYDTEFDVI[M](http://web.expasy.org/cgi-bin/translate/dna_sequences?/work/expasy/tmp/http/seqdna.29744,3,157)TGCGNGSLIE[M](http://web.expasy.org/cgi-bin/translate/dna_sequences?/work/expasy/tmp/http/seqdna.29744,3,168)LQHLNISVDADIVTAYREDTKYILQEVYNFGKVQGGDLIIHEIGSWXPDNEFNEYKYYRRWDFQNIS[M](http://web.expasy.org/cgi-bin/translate/dna_sequences?/work/expasy/tmp/http/seqdna.32220,2,243)K[M](http://web.expasy.org/cgi-bin/translate/dna_sequences?/work/expasy/tmp/http/seqdna.32220,2,245)ILVVQPVPHHFDPESLLGLETVPGVA[M](http://web.expasy.org/cgi-bin/translate/dna_sequences?/work/expasy/tmp/http/seqdna.32220,2,272)ITQTSANVLYAVAK[M](http://web.expasy.org/cgi-bin/translate/dna_sequences?/work/expasy/tmp/http/seqdna.32220,2,287)HNIRTRY

>SlitIR2

GVVQNGGYAYHTEVNSANALISRTFTQSDLCELXSTQS[M](http://web.expasy.org/cgi-bin/translate/dna_sequences?/work/expasy/tmp/http/seqdna.32754,2,43)EKTVLYPCLQKHSPYKEF[M](http://web.expasy.org/cgi-bin/translate/dna_sequences?/work/expasy/tmp/http/seqdna.32754,2,62)TWSL[M](http://web.expasy.org/cgi-bin/translate/dna_sequences?/work/expasy/tmp/http/seqdna.32754,2,67)RLSEQGIVSCIQIRRSSFEVKCEGSSPRPLALGGAAPAFILLAGGYILATII[M](http://web.expasy.org/cgi-bin/translate/dna_sequences?/work/expasy/tmp/http/seqdna.32754,2,120)LVERVIFK[M](http://web.expasy.org/cgi-bin/translate/dna_sequences?/work/expasy/tmp/http/seqdna.32754,2,129)KHANEIPI

>SlitIR4

PLSPXXWWSVIGVSGLCAVLLLLSASFEHRPSSVQYAVFSVVASICQQFFQDXDDGGTKRISTARKATXLVTGLSCVLLYNYYTSSVVSWLLNGPPPSINSLKELXESPLELIYEDIGYTRSWLQTPTYYFNGKNAAIEDEVRKKK

>SlitIR64a

RLLVKLSWTCD[M](http://web.expasy.org/cgi-bin/translate/dna_sequences?/work/expasy/tmp/http/seqdna.1018,1,46)GALICQQGLSINRESTSTKLVIFTTFVYAVTLYQYYNATIVSSLLLEPPRNIRTLKDILDSDLKAGAHDIVYDRDYFKRTTDPVAIELYHKKVATATHYNFFTPEEGIALVKKGGFAFHIDTTFAFPLIKATFTEREICETTLVQ[M](http://web.expasy.org/cgi-bin/translate/dna_sequences?/work/expasy/tmp/http/seqdna.1018,2,192)YPLQR[M](http://web.expasy.org/cgi-bin/translate/dna_sequences?/work/expasy/tmp/http/seqdna.1018,2,198)GVVVRKHSPYKEHIAYAIRK[M](http://web.expasy.org/cgi-bin/translate/dna_sequences?/work/expasy/tmp/http/seqdna.1018,2,219)YEVGLPPRIQSEIDEP[M](http://web.expasy.org/cgi-bin/translate/dna_sequences?/work/expasy/tmp/http/seqdna.1018,2,236)PECAHTPDSSIFVSALE
