# Supplementary material for: A Comparison of the Olfactory Gene Repertoires of Adults and Larvae in the Noctuid Moth Spodoptera littoralis
Source: PLoS One. 2013 Apr 2;8(4):e60263. doi: 10.1371/journal.pone.0060263 (PMC3614943; doi:10.1371/journal.pone.0060263)
Supplement: Supporting Information S4 — Representative RT-PCR amplifications of OBP/CSP transcripts showing differential expression between sexes or developmental stages. (DOCX) [file pone.0060263.s004.docx]

**Supporting information S4**

**Representative RT-PCR amplifications of OBP/CSP transcripts showing differential expression between sexes or developmental stages.**
